# Multivariate Evaluation of Biofunctional Metabolites in Korean Soybean Cultivars by Use Categories: Assessment of Antioxidant and Enzyme Inhibition Activities

**DOI:** 10.3390/antiox14060683

**Published:** 2025-06-04

**Authors:** Kye Man Cho, Se Hyeon Jeon, Eun Jeong Ko, Dong Hyun Park, Ye Ri Jeong, Du Yong Cho, Jeong Ho Kim, Jin Hwan Lee

**Affiliations:** 1 Department of GreenBio Science, Agri-Food Bio Convergence Institute, Gyeongsang National University, Jinju 52727, Republic of Korea; kmcho@gnu.ac.kr (K.M.C.); endyd6098@gnu.ac.kr (D.Y.C.); 2 Department of Smart Green Resources, Dong-A University, 37, Nakdong-daero 550, Saha, Busan 49315, Republic of Korea; bbk6567@naver.com (S.H.J.);; 3 Bio-Resource Research Division, Nakdonggang National Institute of Biological Resources, Sangju 37242, Republic of Korea; rwg2610@nnibr.re.kr

**Keywords:** *Glycine max* (L.), isoflavone, usage category, UPLC-Q-TOF-MS/MS, radical scavenging, DNA protection, enzyme inhibition

## Abstract

This research investigates the metabolite composition and biofunctional activiteies of 41 Korean soybeans, categorized by application: bean sprout, bean paste, vegetable, and cooked-with-rice. Isoflavones were identified via UPLC-Q-TOF-MS/MS and quantified using HPLC, revealing malonylgenistin as the predominant composition (average 743.4 μg/g, 42.3% of total isoflavones). Bean sprout showed the highest average isoflavone (2780.6 μg/g), followed by bean paste (1837.8 μg/g), cooked-with-rice (1448.2 μg/g), and vegetable (883.2 μg/g), with significant differences in individual cultivars. Protein ranged from 36.8 to 46.6% and oil from 17.0 to 22.3%, with vegetable soybeans exhibiting the highest average protein (44.9%) and lowest average oil (18.6%). Moreover, PLS-DA and hierarchical clustering revealed distinct metabolic patterns in usage groups. Antioxidant activities (radical scavenging; DNA protection) and enzyme inhibition (tyrosinase; α-glucosidase) also varied significantly, correlating with isoflavone distributions. Particularly, Sorog exhibited the highest isoflavone (3722.7 μg/g) and strong antioxidant activity (DPPH: 72.2%; ABTS: 93.8%, 500 μg/mL), DNA protection (92.8%, 200 μg/mL), and inhibition of tyrosinase and α-glucosidase by 78.4% and 84.2% (500 μg/mL). These findings suggest that isoflavone-rich bean sprout soybeans, especially Sorog, are promising candidates for health-promoting foods and functional cultivar development. This is the first systematic study comparing the metabolites and health-related properties of soybeans based on Korean usage categories.

## 1. Introduction

Natural plants, such as crops, vegetables, fruits, and cereal sources, are subjected to complex interactions from various environmental factors [1,2,3,4,5], and exposure to both biotic and abiotic stresses disrupts their metabolic processes, leading to slower growth and reduced productivity [2,5,6,7]. Plants generally use signaling pathways to modulate their transcriptome and metabolomic profiles in response to stress [5,7]. In particular, transcription factors regulate plant defense mechanisms, including the synthesis of phytohormones, increased metabolite formation, osmotic regulation to maintain growth and productivity, and reactive oxygen species signaling [8]. Recent studies have shown that the complex signaling pathways and functions of primary and secondary metabolites in stress responses provide valuable insights for developing strategies to improve plant resilience, agricultural productivity, and sustainability [5,7]. Numerous researchers have investigated metabolite constituents in diverse plants because of their health functions and pharmacological properties (antioxidant, anticancer, anti-diabetic, anti-atherosclerotic, anti-inflammatory, etc.) during the past years [9,10,11,12,13,14]. In addition, a wide range of natural plant-based food products have been developed, and their global consumption continues to increase. It is well known that primary metabolites are essential for plants’ physiological processes, contributing significantly to growth and development [5,15]. These include carbohydrates, proteins, and lipids, which are essential for physiological functions, serve as precursors for macromolecule synthesis, and provide energy for plant development [15,16,17,18]. Specifically, it has been reported that the content of protein and oil, alongside anti-nutritional factors and the activities of enzymes that regulate these functions, exhibit substantial variability among soybean varieties, which significantly affects digestibility and bioavailability [18,19,20]. Another class of constituents, secondary metabolites, contributes to plant sustainability [1,5] and is classified based on chemical structures such as flavonoids, terpenes, polysaccharides, alcohols, and phenolic acids [6,9,10,14,21,22]. Due to their potent functional properties and health benefits, these compounds are gaining increasing attention in food and medicinal applications [11,12,14,18,23]. These metabolites are widely abundant in various crops.

It is commonly demonstrated that soybeans (*Glycine max* (L.)) are one of the most valuable crops, owing to the presence of many metabolites and their nutraceutical abilities [9,13,17,18,24,25]. In particular, isoflavones have been identified as the most important classes in terms of their functional and pharmacological characteristics [9,26,27,28]. The soybean isoflavones are recognized as 12 components divided into four types, with their typical distribution being malonylglucosides (70–80%), glucosides (25%), acetylglucosides (5%), and aglycones (2%) [4,19]. Furthermore, this constituent is renowned for its health benefits, such as the reduction of cancers, cardiovascular diseases, and diabetes with antimicrobial, antioxidant, and antiviral properties [19,24,28,29]. It had previously also been documented that this species is also rich in metabolites, namely, protein, oil, terpene, saponin, and phenolic acid, except isoflavone [9,13,17,24,30]. Interestingly, soy food products in the Republic of Korea have been widely produced as cooking soybeans (*Cheonggukjang*), soybean cake (*Meju*), soybean paste (*Doenjang*), and soybean sauce (*Kanjang*) using different conditions and microorganism strains [31,32]. The optimal fermentation conditions differ depending on the soybean cultivar, which are typically categorized by their intended applications: bean paste, bean sprout, vegetable, or cooked-with-rice types [33]. Given these factors, soybeans are of increasing interest to the functional food and dietary supplement industries. Crop breeding programs have particularly emphasized the development of soybean cultivars possessing high metabolite contents and strong bioactive values. Although numerous studies have documented that the concentration and distribution of metabolites exhibit significant variations among soybean cultivars under various conditions [9,24,25,28,34], there is currently a lack of comprehensive information regarding the determination and comparison of antioxidant effects, enzyme inhibition properties, and metabolite contents across new cultivars. This information is essential for the selection of superior sources to facilitate the development of functional food products and new cultivars. Furthermore, few systematic studies have assessed metabolite diversity and biofunctionality in soybeans based on their categorized uses. Accordingly, our work was designed to gather information regarding the comparisons and patterns of metabolite constituents as well as their biofunctional abilities in various Korean soybean cultivars classified by their intended applications.

The primary objective of this research was to identify excellent Korean soybean cultivars that possess not only high metabolite contents but also strong biological effects, facilitating the development of new functional agents. Herein, the presence of 12 isoflavone derivatives of the most functional constituents of soybeans was primarily verified in a 50% methanol extract using UPLC-ESI-Q-TOF-MS/MS analysis. Additionally, our study was the first to compare and assess metabolite contents from various soybean cultivars categorized by their intended applications, such as isoflavone, protein, and oil. We specifically evaluated the correlations between metabolite constituents and soybean types, focusing on their interaction relationships by partial least squares discriminant analysis (PLS-DA) and heatmap analysis. The present research also documents the antioxidant properties, including radical scavenging and DNA protection, as well as the inhibition capacities of tyrosinase and α-glucosidase.

## 2. Materials and Methods

### 2.1. Plant Sources and Chemicals

Forty-one soybean cultivars, developed by the National Institute of Crop Science (NICS) Rural Development Administration (RDA) in the Republic of Korea, were classified into four groups: bean paste, bean sprout, vegetable, and cooked-with-rice. Detailed information is included at https://www.nics.go.kr/oneStopIndex (accessed on 19 May 2025). All the cultivars were harvested from experimental fields (NICS, Milyang, Gyeongnam-do, Republic of Korea). Seeds were sown on 15 October 2022 and harvested between 3:00 and 5:00 p.m. Harvested seeds were immediately washed with sterile water, air-dried at room temperature for 3 days, and stored at −10 °C prior to analysis. 2,2-Diphenyl-1-pycrylhydrazyl (DPPH), 2,2′-azino-bis(3-ethylbenzthiazoline-6-sulphonic acid) (ABTS), 6-hydroxy-2,5,7,8-tetramethylchroman-2-carboxylic acid (Trolox), butylated hydroxytoluene (BHT), tyrosinase (EC 1.14.18.1), α-glucosidase (EC 3.2.1.20), ascorbic acid, acarbose, and trifluoroacetic acid (TFA) were obtained from Sigma-Aldrich Chemical Co. (St. Louis, MO, USA). Dithiothreitol (DTT), FeCl_3_, L-tyrosine, *p*-nitrophenyl-α-D-glucopyranoside (PNP-G), and *p*-nitrophenol (*p*-NP) were also acquired from Sigma-Aldrich. The super-coiled plasmid DNA pUC18 was provided by Thermo Fisher Scientific (Waltham, MA, USA). All other chemicals and reagents were of analytical grade and provided by Sigma-Aldrich. HPLC solvents (water and methanol) were obtained from J.J. Baker (Phillipsburg, NJ, USA). Standard isoflavone glucosides were isolated from a specific soybean cultivar (cv. Sorog) following previously described methods [24,35] with minor modifications. Acetyl-, malonyl-, and aglycone isoflavones were purchased from Sigma-Aldrich (St. Louis, MO, USA).

### 2.2. Instruments

The ^1^H- and ^13^C-NMR spectra used to confirm the isolated isoflavone glucosides were measured using a Bruker AM 500 (^1^H NMR at 500 MHz, ^13^C NMR at 125 MHz) spectrometer (Bruker, Germany) in CF_3_COOD-CD_3_OD (1/19, *v*/*v*). Antioxidant activities against radicals (DPPH and ABTS) and enzyme inhibition effects (tyrosinase and α-glucosidase) were investigated using UV–Vis spectrophotometry with an Agilent BioTek microplate reader (EPOCH 2, Winooski, VT, USA). DNA protection rates were evaluated by the Gel Doc XR+ system (Bio-Rad, Hercules, Contra Costa, CA, USA). HPLC analysis was performed using an Agilent 1100 liquid chromatograph system (Agilent Technologies Co., Santa Clara, CA, USA) equipped with a quaternary pump, vacuum degasser, autosampler, UV detector, and thermal chamber controller. A reversed-phase column [LichroCART 125-4 HPLC-Cartridge (Lichrophore 100 RP-18e) (125 m × 4 mm i.d., 5 μm, Merck KGaA, Darmstadt, Germany)] was used for the separation. Protein and oil contents were determined using a B-339 Auto Kjeldahl analyzer (Büchi Labortechnik AG, Flawil, Schweiz) and a BÜCHI B-811 Extraction System (Büchi, Schweiz). Moreover, the chemical structures of 12 isoflavones in the soybean extract were elucidated by a UPLC system (1290 Infinity II LC, Agilent Technologies Co., Santa Clara, CA, USA) coupled with a quadrupole time-of-flight mass spectrometer (Agilent 6546 LC/Q-TOF, Agilent Technologies Co., Santa Clara, CA, USA) operating in positive electrospray ionization mode.

### 2.3. Antioxidant Properties of Radical Scavenging Capacities

To measure radical scavenging activity, pulverized soybean seeds (1.0 g, 60 mesh) were extracted with 20 mL of 50% methanol for 3 days at room temperature. The crude extract was filtered through Whatman paper (No. 42 filter), and the resulting supernatants were used for further analysis. Radical scavenging assays were conducted to analyze the antioxidant effect, following previously reported methods with slight modifications [24]. A 1 mM DPPH solution was prepared and adjusted to an absorbance of 0.70 at 517 nm. To this solution, 100 μL of the 50% methanol extract (from soybean seeds) or 100 μL of BHT (positive control) at various concentrations was added to the DPPH solution (3.9 mL) [28]. The resulting mixture was maintained for 30 min at 25 °C in the dark, and the absorbance value was measured at 517 nm. The DPPH radical scavenging capacity was demonstrated as a percentage using the following formula:

Scavenging effect (%) = (1 − absorbance of sample/absorbance of the control) × 100
(1)

The ABTS radical scavenging ability was assessed using the method described by Lee et al. [22] and Hwang et al. [24]. This assay evaluated the ability of different substances to scavenge the ABTS^•+^ by comparing their effects to that of Trolox (positive control). For the ABTS method, the reaction mixture consisted of 7 mM ABTS (dissolved in ethanol) and 2.45 mM potassium persulfate. This solution was kept in the dark at 25 °C for 12 h. The ABTS^•+^ solution was then diluted with ethanol to achieve an absorbance of 0.70 ± 0.02 at 734 nm. After adding the ABTS^•+^ solution (0.9 mL) to the soybean extract (0.1 mL), the absorbance value at 734 nm was recorded. The scavenging capacity was calculated as a percentage using the following equation:

Scavenging capacity (%) = [(absorbance of control − absorbance of sample)/absorbance of control] × 100
(2)

### 2.4. Measurement of DNA Protection Rate

To assess the DNA protection rate of the 50% methanol extract of soybean seeds, the metal-catalyzed oxidation (MCO) DNA cleavage assay was performed as previously described [19,23,36], with slight modification. Supercoiled plasmid DNA (pUC18 from E. coli) was prepared at 50 μg/mL in phosphate-buffered saline (0.5 M, pH 7.4). The extracts of soybean seeds at various concentrations (10, 20, 50, 100, 200, 500 μg/mL, 5 μL) were combined with supercoiled plasmid DNA (5 μL), dithiothreitol (3.3 mM, 5 μL), and FeCl_3_ (15.4 μM, 5 μL) and incubated for 2 h at 37 °C. A portion of the reaction solution (5 μL) was mixed with a DNA loading buffer (1 μL) and then placed onto a 0.8% agarose gel in a Tris-acetate EDTA buffer, which had Tris-acetate (40 mM) and EDTA (1 mM). After electrophoresis (30 min at 85 V), DNA bands were visualized and photographed under UV light using the Gel Doc XR+ system (Bio-Rad, Hercules, Contra Costa, CA, USA). DNA band intensities were analyzed using Image Lab software version 6.1 (Bio-Rad, Hercules, Contra Costa, CA, USA), and DNA protection (%) was calculated using the following formula [23]:

DNA band protection (%) = (SF DNA band intensity/pUC18 plasmid DNA band intensity) × 100
(3)

### 2.5. Determination of Enzyme Inhibition Effects

To determine the enzyme inhibition abilities of different soybean categories, tyrosinase and α-glucosidase assays were performed using previously described spectrophotometric techniques [9,14,34]. The soybean extracts used in these assays were prepared following the same protocol as for antioxidant analysis. The tyrosinase inhibitory effect was demonstrated with L-DOPA as a substrate, according to the method reported by Solimine et al. [11] and Zheng et al. [14], with slight modifications. The reaction mixture consisted of 685 μL of 0.1 M NaH_2_PO_4_ buffer (pH 6.8), 20 μL of soybean extract at various concentrations, 15 μL of tyrosinase (250 unit/mL), and 100 μL of 5 mM L-DOPA. The mixture was incubated at 37 °C for 25 min, and the absorbance was measured at 475 nm using a UV–Vis spectrophotometer. Ascorbic acid was used as a positive control. The percentage of inhibition was calculated by the following formula:

Inhibition rate (%) = [(A control − A sample)/A control] × 100
(4)

A control = the absorbance of buffer. A sample = the absorbance of the reaction solution containing sample extract.

The α-glucosidase inhibitory effect was determined following the methods of Chen et al. [9] and Kim et al. [34], with acarbose as a positive control. A mixture of 50% methanol extract (100 μL) and 0.1 M phosphate buffer (pH 7.0, 100 μL) containing α-glucosidase (1.0 U/mL) was prepared in a quartz cuvette. After pre-incubation for 10 min at 25 °C, 50 mM *p*-nitrophenyl-α-D-glucopyranoside (PNP-G) (50 μL) was added. A spectrophotometer analyzed the enzymatic solution by measuring the release of p-nitrophenol from PNP-G at 405 nm. One unit of α-glucosidase is defined as the amount of enzyme that produces 1.0 μM of PNP/min. The inhibitory ability was assessed as a percentage (%) using the following procedure:

α-Glucosidase inhibitory ability (%) = [(A−B/A)] × 100
(5)

where A represents the absorbance of the mixture containing (0.1 M phosphate buffer + 50 mM PNP-G solution + α-glucosidase solution), and B is the absorbance of the mixture containing (extract of soybean seeds + 50 mM PNP-G solution + α-glucosidase solution).

### 2.6. UPLC-Q-TOF-MS/MS Conditions for Isoflavone Identification

The 50% methanol extract of cultivated soybean was analyzed using a UPLC-Q-TOF-MS/MS system for the identification of isoflavones [34,37]. The UPLC system was equipped with a vacuum degasser, a binary pump, and automatic sampler and coupled to a quadrupole time-of-flight mass spectrometer operating in positive electrospray ionization mode. A sample (5 μL) was injected into an analytical reverse phase C18 column (Phenomenex Kinetex EVO C18, 100 mm × 2.1 mm, 2.6 μm, Torrance, CA, USA) with a flow rate of 0.2 mL/min. The mobile phase consisted of solvent A (water) and solvent B (0.1% formic acid in CH_3_CN), with the gradient conditions of 0–2 min, 3% B; 10 min, 10% B; 30 min, 40% B; 40 min, 95% B, and was then held for 3 min before returning to the initial conditions. The column temperature was set at 25 °C. Mass spectrometric analysis was performed as *m*/*z* 0–600. The detailed mass spectrometry conditions were as follows: ion source, Dual AJS ESI; gas temperature, 300 °C; gas flow, 9 L/min; nebulizer pressure, 35 psi; sheath gas temperature, 350 °C; sheath gas flow, 11 L/min; capillary voltage, 4000 V; nozzle voltage, 500 V; octupole RF, 750 V; fragmentor voltage, 175 V; skimmer voltage, 45 V; MS scan rate, 5 spectrals; MS/MS scan rate, 3 spectrals; and collision energy, 40 eV. Data acquisition, peak integration, and isoflavone identification were conducted using MassHunter Qualitative Analysis Software B 8.00 (Agilent Technologies, Co., Santa Clara, CA, USA).

### 2.7. Measurement of Protein and Oil Contents

To evaluate the protein content, the powdered seeds (0.2 g) were processed using a Buchi B-435 digestion system, which involved the addition of 20 mL of H_2_SO_4_ and 3.0 g of catalyst (K_2_SO_4_:CuSO_4_ = 9:1). This apparatus was connected to a scrubber and employed the Kjeldahl nitrogen method in a distillation unit [19]. For the quantification of oil, the Soxhlet extraction method was utilized with the Buchi B-811 extraction system [19]. In this procedure, the pulverized soybean seeds (2.0 g) were placed in an extraction thimble containing 200 mL of hexane and heated at 105 °C for 2 h. The weight of the extracted oil was measured and reported as a percentage of the dry matter of the soybean seeds.

### 2.8. Preparation of Samples and Calibration Curves for Quantification of Isoflavones

The preparation of the sample and the construction of calibration curves were conducted as described in the previous literature [24,35]. The dried soybean seeds were ground with an HR 2860 coffee grinder (Koninklijke Philips N.V., Amsterdam, The Netherlands) for 3 min. The pulverized seeds (1.0 g) were extracted using 20 mL of 50% methanol for 12 h at 25 °C in a shaking incubator. The crude solution was subjected to centrifugation at 3000× *g* for 3 min and then passed through a 0.45 μm syringe filter (Whatman Inc., Maidstone, UK). The filtered supernatant was stored at 4 °C before HPLC analysis. The stock solution of the isoflavone standard was prepared with DMSO to obtain a concentration of 1000 μg/mL. The peak areas for each standard were integrated from the HPLC chromatograms at 254 nm and plotted against their concentrations to generate a linear curve. Calibration curves were developed at eight concentration levels (1, 5, 10, 20, 40, 80, 100, and 200 μg/mL) by diluting each stock solution with DMSO. Each isoflavone curve exhibited high linearity, with *r*^2^ values greater than 0.999.

### 2.9. HPLC Conditions for Isoflavone Analysis

Isoflavone content was analyzed using HPLC according to the method described by Hwang et al. [24] and Lee et al. [35], with a minor modification. The total run time was 40 min at a flow rate of 1.0 mL/min, with the column temperature maintained at 25 °C. The mobile phase consisted of 0.1% acetic acid (*v*/*v*) in water (eluent A) and acetonitrile (eluent B). The gradient was programmed as, from 0 to 20 min, 20% B; at 30 min, 25% B; at 40 min, 35% B, followed by a hold for 5 min before returning to the initial conditions. A 20 μL sample of the crude extract was injected into an analytical C18 column (Lichroprep 100 RP-18, 5 μm, 125 mm × 4 mm, Merck KGaA, Darmstadt, Germany), with detection occurring at 254 nm.

### 2.10. Statistical Analysis

The isoflavone, protein, oil contents, antioxidant, and enzyme inhibition properties were presented as the mean ± standard deviation (SD) of triplicate measurements. To analyze the differences among these values, Tukey’s HSD-ANOVA multiple range test was employed at a significant level of 0.05, using the SAS 9.2 software package (SAS Institute Inc., Cary, NC, USA). PLS-DA was executed with R Project (version 4.5.0, Vienna, Austria), producing score plots for Comp1 and Comp2 within 95% confidence intervals. Heatmaps were generated based on Pearson correlation distance and Ward’s clustering method, following established protocols in the literature [36,38].

## 3. Results

### 3.1. Comparisons of Antioxidant Abilities in Four Categories Based on Usage from Various Cultivated Soybeans

**Figure 1:**
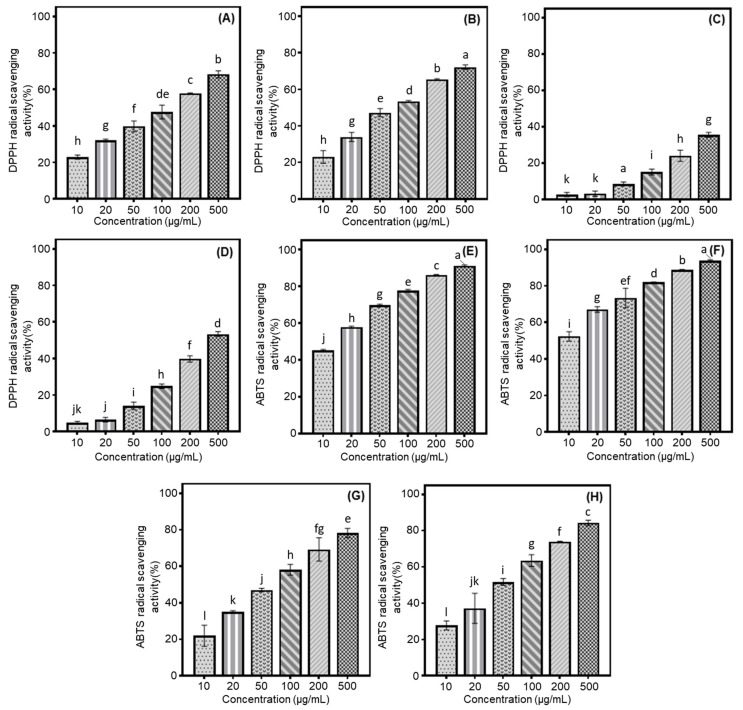
Comparison of soybean cultivars with the radical scavenging activity of four usage categories using different concentrations of 50% methanol extracts. (**A**) DPPH radical scavenging effects of bean paste soybean (cv. Shinpaldal 2); (**B**) DPPH radical scavenging effects of bean sprout soybean (cv. Sorog); (**C**) DPPH radical scavenging effects of vegetable soybean (cv. Hwaeomput); (**D**) DPPH radical scavenging effects of cooked-with-rice soybean (cv. Cheongdu 1); (**E**) ABTS radical scavenging effects of bean paste soybean (cv. Shinpaldal 2); (**F**) ABTS radical scavenging effects of bean sprout soybean (cv. Sorog); (**G**) ABTS radical scavenging effects of vegetable soybean (cv. Hwaeomput); (**H**) ABTS radical scavenging effects of cooked-with-rice soybean (cv. Cheongdu 1). All values are expressed as the mean ± standard deviation of triplicate measurements, and different letters in the graph indicate significant differences at the 0.05 level of Tukey’s HSD-ANOVA multiple range test between usage categories to concentrations (*p* ≤ 0.05).

Our study examined the antioxidant properties of cultivated soybeans using DPPH and ABTS radical scavenging assays, focusing on different categories based on their intended usage. Their activities were evaluated based on the percentage inhibition of radical formation by the seed extracts or positive controls (DPPH: BHT and ABTS: Trolox). Building on previous results and preceding experiments, we assessed the radical scavenging effects at various concentrations (2000, 1500, 1000, 500, 250, and 100 μg/mL) to determine the optimal conditions. Although 100% radical inhibition was observed at concentrations of 2000, 1500, and 1000 μg/mL, the current research was conducted at 500 μg/mL due to dose-dependent variations in inhibition rates. Especially, the rank order of scavenging abilities of two radicals was observed to increase as follows: bean sprout > bean paste > cooked-with-rice > vegetable soybeans (Figure 1A–H). This sequence of radical scavenging activities was consistent with the average isoflavone contents measured in each sample, which were as follows: bean paste, 1837.8 μg/g; bean sprout, 2780.6 μg/g; vegetable, 883.2 μg/g; and cooked-with-rice, 1448.2 μg/g (Table 1). In various samples of bean paste and bean sprout soybeans, the Shinpaldal 2 and Sorog cultivars exhibited the highest DPPH radical scavenging activities, with considerable changes through different concentrations (500 → 10 μg/mL), as detailed below: 68.3 → 57.9 → 47.7 → 39.8 → 32.2 → 22.9% (Shinpaldal 2, Figure 1A); 72.2 → 65.4 → 53.4 → 47.3 → 34.0 → 23.0% (Sorog, Figure 1B). The Hwaeomput and Cheongdu 1 cultivars also showed notable effects in vegetable and cooked-with-rice sources, with the increase rates of soybean extracts as follows: 35.5 → 24.1 → 15.0 → 8.5 → 3.2 → 2.7% (Hwaeomput, Figure 1C); 53.4 → 39.7 → 24.9 → 14.2 → 6.6 → 4.8% (Cheongdu 1, Figure 1D). Their DPPH radical scavenging effects increased with rising extract concentrations (10 → 500 μg/mL), showing statistically significant differences. To summarize, the scavenging activities against DPPH radicals in four different categories of soybeans were ranked in the following order: Sorog > Shinpaldal 2 > Cheongdu 1 > Hwaeomput. These results were consistent with their respective isoflavone contents, showing a similar decreasing trend: 3389.4 μg/g (Sorog) > 2825.4 μg/g (Shinpaldal 2) > 2205.5 μg/g (Cheongdu 1) > 1102.3 μg/g (Hwaeomput). The radical scavenging activity of each variety was quantified compared to the positive control, BHT. There was a significant difference among the four soybean varieties, with Sorog (101.27 mg/g) showing the highest activity, followed by Shinpaldal 2 (97.55 mg/g), Cheongdu 1 (75.45 mg/g), and Hwaeomput (51.17 mg/g) (Table S1). In the ABTS radical method, the extracts showed similar trends to the DPPH radical inhibition results, with increasing concentrations from 10 to 500 μg/mL. The extent of these scavenging effects, however, varied considerably in the cultivars and their applications within the 10 to 500 μg/mL concentration range, as follows: Sorog (52.4 → 93.8%, Figure 1F; bean sprout) > Shinpaldal 2 (45.2 → 91.3%, Figure 1E; bean paste) > Cheongdu 1 (27.7 → 84.3%, Figure 1H; cooked-with-rice) > Hwaeomput (21.9→ 78.3%, Figure 1E; vegetable). Moreover, the values were measured based on the positive control, trolox. Sorog (181.55 mg/g) showed the highest activity, followed by Shinpaldal 2 (169.72 mg/g), Cheongdu 1 (126.31 mg/g), and Hwaeomput (117.39 mg/g). These trends demonstrate the excellent ABTS radical scavenging ability of sprouts and paste beans (Table S1).

### 3.2. Comparisons of DNA Protection Rates in Four Categories Based on Usage from Various Cultivated Soybeans

**Figure 2:**
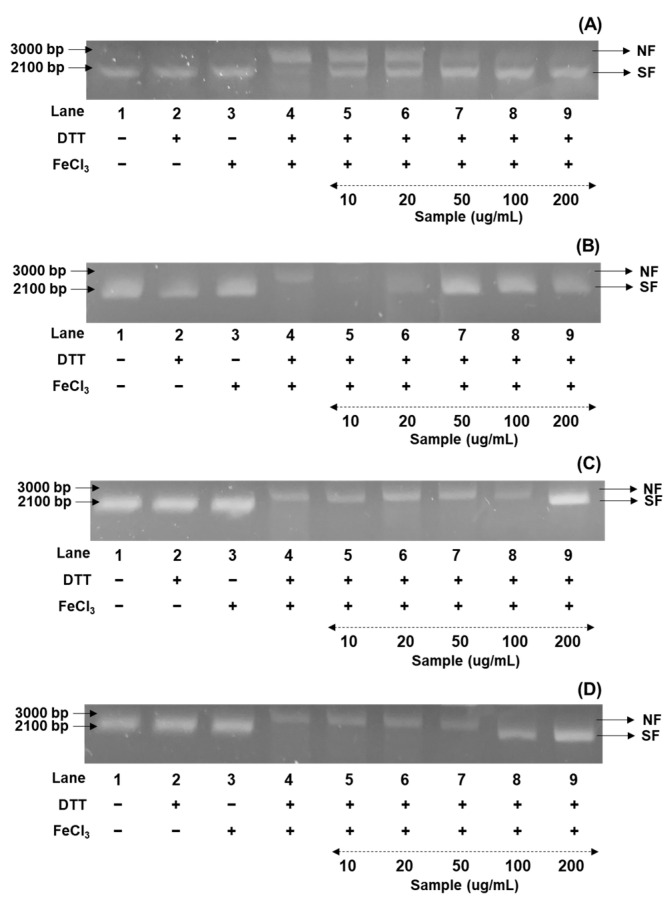
Comparison of soybean cultivars with the DNA protectant effects of four usage categories using different concentrations of 50% methanol extracts. (**A**) Bean paste soybean (cv. Shinpaldal 2); (**B**) bean sprout soybean (cv. Sorog); (**C**) vegetable soybean (cv. Hwaeomput); and (**D**) cooked-with-rice soybean (cv. Cheongdu 1). Lane 1, pUC18 only; lane 2, pUC18 with DDT only; lane 3, pUC18 with FeCl_3_ only; lane 4, pUC18 with MCO system; lane 5–9, pUC18 with combinant extracts in the MCO system (lane 5: 10 μg/mL; lane 6: 20 μg/mL; lane 7: 50 μg/mL; lane 8: 100 μg/mL; and lane 9: 200 μg/mL).

In the present research, the DNA damage protection ability of soybeans was investigated against hydroxyl radicals generated with nicked DNA pUC18, using gel electrophoresis [36]. In the present research, the DNA protection assay was examined as percentage values as five concentrations (10, 20, 50, 100, and 200 μg/mL), based on the results of radical inhibition experiments, and were compared with the percentage protections of the control from the 50% methanol extracts of cultivated soybeans. Their protection patterns were consistent with the radical scavenging abilities, as the order of bean sprout > bean paste > cooked-with-rice > vegetable soybeans. Although the DNA protection rates varied considerably in diverse soybean cultivars, the highest protective bands were consistently observed in four representative cultivars based on their categorized applications (Figure 2A–D). We characterized the differences in DNA protection for the Shinpaldal 2, Sorog, Hwaeomput, and Cheongdu 1 cultivars, which showed the strongest radical scavenging effects within each soybean group. As illustrated in Figure 2A–D, the DNA protective abilities displayed remarkable differences with concentration-dependent changes in each extract at the five concentrations. The Sorog cultivar showed the highest protection rates with 66.5 → 83.5 → 89.7 → 92.8% in the range of 20 → 200 μg/mL (Figure 2A), while the lowest ratios were detected with protection rates of 18.0, 22.2, 28.3, and 79.6% at 20, 50, 100, and 200 μg/mL in Hwaeomput (Figure 2B). The remaining cultivars were observed as, in decreasing order, Shinpaldal 2 (56.3 → 76.2 → 80.7 → 87.5%) (Figure 2C) > Cheongdu 1 (18.4 → 20.6 → 75.7 → 80.7%) (Figure 2D), with clear differences depending on extract concentrations. Furthermore, the 50% methanol extracts of the four cultivars provided DNA protection even at the lowest concentration (10 μg/mL), with values of 44.5% (Shinpaldal 2), 24.6% (Sorog), 11.5% (Hwaeomput), and 16.6% (Cheongdu 1), compared to the control. Interestingly, although the Sorog cultivar showed the most protective capacities, its protection rate sharply declined from 66.5% to 24.6% between 10 and 20 μg/mL, whereas Shinpaldal 2 displayed a milder reduction (56.3% → 44.5%). These DNA protection trends were consistent with the radical inhibition patterns.

### 3.3. Comparisons of Enzyme Inhibition Activities on Tyrosinase and α-Glucosidase in Four Categories Based on Usage from Various Cultivated Soybeans

**Figure 3:**
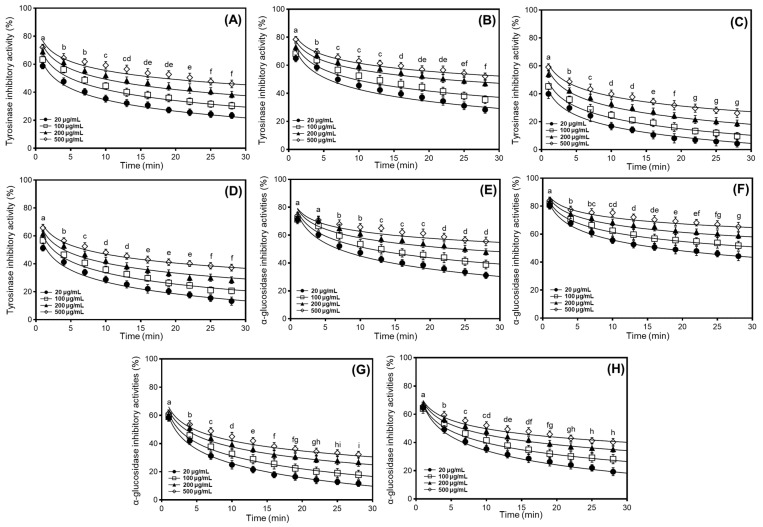
Comparison of soybean cultivars with the highest enzyme inhibitory properties, including tyrosinase and α-glucosidase in four usage categories using different concentrations of 50% methanol extracts. (**A**) Tyrosinase inhibition effects of bean paste soybean (cv. Shinpaldal 2); (**B**) tyrosinase inhibition effects of bean sprout soybean (cv. Sorog); (**C**) tyrosinase inhibition effects of vegetable soybean (cv. Hwaeomput); (**D**) tyrosinase inhibition effects of cooked-with-rice soybean (cv. Cheongdu 1); (**E**) α-glucosidase inhibition effects of bean paste soybean (cv. Shinpaldal 2); (**F**) α-glucosidase inhibition effects of bean sprout soybean (cv. Sorog); (**G**) α-glucosidase inhibition effects of vegetable soybean (cv. Hwaeomput); and (**H**) α-glucosidase inhibition effects of cooked-with-rice soybean (cv. Cheongdu 1). All values are expressed as the mean ± standard deviation of triplicate measurements, and different letters in the graph indicate significant differences at the 0.05 level of Tukey’s HSD-ANOVA multiple range test between times (*p* ≤ 0.05).

We investigated the tyrosinase and α-glucosidase inhibitory properties of different soybean cultivars, comparing the percentage inhibition ratios according to their applications. Based on preliminary findings and the antioxidant effects observed in radical scavenging and DNA protection assays, we evaluated the tyrosinase and α-glucosidase inhibitory characteristics of 50% methanol extracts from cultivated soybeans, which exhibited dose-dependent inhibition at concentrations ranging from 20 to 500 μg/mL (Figure 3). In the usage classifications of soybean cultivars, the two enzyme inhibitory capacities varied significantly between cultivars and their applications, following the order bean sprout > bean paste > cooked-with-rice > vegetable. To further elucidate the enzyme inhibitory properties, we characterized individual soybean cultivars (Shinpaldal 2, Sorog, Hwaeomput, and Cheongdu 1) exhibiting the highest isoflavone content and strongest antioxidant activity within each of the four categories. As shown in Figure 3A–D, the tyrosinase inhibitory activities of 50% methanol extracts from four soybean cultivars were evaluated at various concentrations (500, 200, 100, and 20 μg/mL). The inhibition rates showed considerable differences with concentration-dependent variations, and the rank order of inhibitory activity at 20–500 μg/mL, similar to the antioxidant activities, was Sorog > Shinpaldal 2 > Cheongdu 1 > Hwaeomput. Under a 30 min enzyme reaction at 500 μg/mL, the Sorog cultivar exhibited the predominant inhibition rates, decreasing from 78.4 to 52.2% (Figure 3B). The inhibition ratios at other concentrations also were observed similar patterns with dose-dependent variations (75.1 → 47.2% at 200 μg/mL; 74.2 → 35.6% at 100 μg/mL; 73.0 → 28.3% at 20 μg/mL). The Shinpaldal 2 cultivar showed the second inhibitory capacity, with clear variations in concentrations (Figure 3A). After 30 min, the inhibition rates at different concentrations (20 to 500 μg/mL) were 66.7% to 23.5% at 20 μg/mL; 69.4% to 30.5% at 100 μg/mL; 71.1% to 38.4% at 200 μg/mL; and 72.1% to 45.9% at 500 μg/mL. The remaining cultivars, Hwaeomput and Cheongdu 1 (Figure 3C,D), exhibited clearly low inhibition effects (Hwaeomput: 59.1 → 26.2% at 500 μg/mL and 55.9 → 4.3% at 20 μg/mL; Cheongdu 1: 65.8 → 37.3% at 500 μg/mL and 61.3 → 13.3% at 20 μg/mL), approximately two times that when compared with Sorog and Shinpaldal 2. Quantitative analysis of 50% methanol extracts, expressed relative to the positive control (ascorbic acid), ranked the cultivars in the following order: Sorog (53.73 mg/g) > Shinpaldal 2 (49.17 mg/g) > Cheongdu 1 (47.58 mg/g) > Hwaeomput (41.75 mg/g) (Table S1). As illustrated in Figure 3E–H, the inhibition patterns against α-glucosidase also exhibited significant differences with the dose-dependent changes in the four cultivars, following a similar trend to tyrosinase inhibition. The rank order of inhibitory activity was Sorog > Shinpaldal 2 > Cheongdu 1 > Hwaeomput. During the enzyme reaction at 500 μg/mL for 30 min, the absolutely highest inhibition activities were detected in the Sorog cultivar with variations of 84.2 → 65.3% (Figure 3F), followed by Shinpaldal 2 (74.1 → 55.5%, Figure 3E) > Cheongdu 1 (66.0 → 40.3%, Figure 3H) > Hwaeomput (61.5 → 32.2%, Figure 3G). Similarly, the quantitative values of 50% methanol extracts for α-glucosidase inhibition, relative to the positive control (acarbose), followed the same descending order: Sorog (52.66 mg/g) > Shinpaldal 2 (44.70 mg/g) > Cheongdu 1 (39.95 mg/g) > Hwaeomput (38.34 mg/g) (Table S1).

### 3.4. Distribution of Protein and Oil Contents in Four Categories Based on the Applications of Various Cultivated Soybeans

In this research, we investigated protein and oil concentrations by comparing their differences in four Korean soybean applications. As illustrated in Table 2, protein content showed slight variations in the four soybean usage categories, with no remarkable differences observed between cultivars. This metabolite content ranged from 38.9 to 44.1% (bean paste), 36.8 to 44.0% (bean sprout), 43.1 to 46.6% (vegetable), and 40.2 to 43.9% (cooked-with-rice), respectively (Table 2). Among the various cultivars, Jangwon, Daehwang, Eunha, Keunol, Hwaeomput, Hwasonput, Seonnog, and Dajin exhibited relatively high protein contents, exceeding 44% compared to other cultivars. Interestingly, vegetable soybeans displayed the highest average protein content at 44.9%, followed by cooked-with-rice soybeans (42.0%), bean paste soybeans (41.2%), and bean sprout soybeans (40.0%). In the bean paste category, Jangwon and Daehwang showed the highest protein contents at 44.1% in the eleven cultivars, while the lowest content was observed in Taekwang (38.9%). The relatively maximum content for protein regarding bean sprout soybeans was detected in the Eunha cultivar, with 44.0%, followed by Sorog (42.6%), Anpyeong (41.7%), Iksannamul (40.9%), and Sobaegnamul (40.1%), compared to other cultivars. Sojin showed the lowest protein content at 36.8%. Notably, the protein content of all the vegetable soybean cultivars was considerably higher (> 43%) than that of other categories (average contents: bean paste, 41.2%; bean sprout, 40.0%; and cooked-with-rice, 42.0%) (Table 2). Specifically, the most abundant protein was observed in the Dajin cultivar (46.6%), followed by Hwaeomput (46.4%) > Hwasonput (45.7%) > Seonnog (44.8%) > Keunol (44.6%), and the remaining cultivars had protein contents exceeding 43%. Cooked-with-rice soybeans also showed slight variations in the eleven cultivars, demonstrating no remarkable differences in the increasing order of Geomjeong 3 (43.9%) > Cheongdu 1 (43.6%) > Seonheuk (43.5%), and, in other cultivars, ranges were detected from 40.2 to 42.5%. The oil content exhibited lower differences compared to protein, and its variations were also slight in cultivars and usage categories (Table 2). In the four different categories of 41 soybean cultivars, the average oil contents were 20.5 (bean paste), 20.4 (bean sprout), 18.6 (vegetable), and 20.2% (cooked-with-rice), respectively. And their contents were measured in the ranges of 18.8–21.9, 18.5–22.3, 17.0–20.2, and 18.6–21.3%, respectively. The highest content was observed in Iksannamul (22.3%), while the lowest was found in Keunol, with 17.0%.

### 3.5. Elucidation of Isoflavone Profiles Using UPLC-ESI-Q-TOF-MS/MS Analysis from the 50% Methanol Extract of Soybean (cv. Sorog)

**Figure 4:**
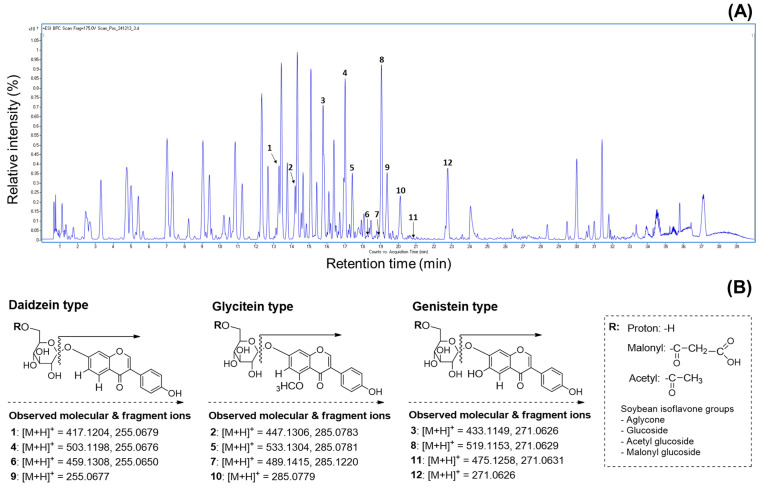

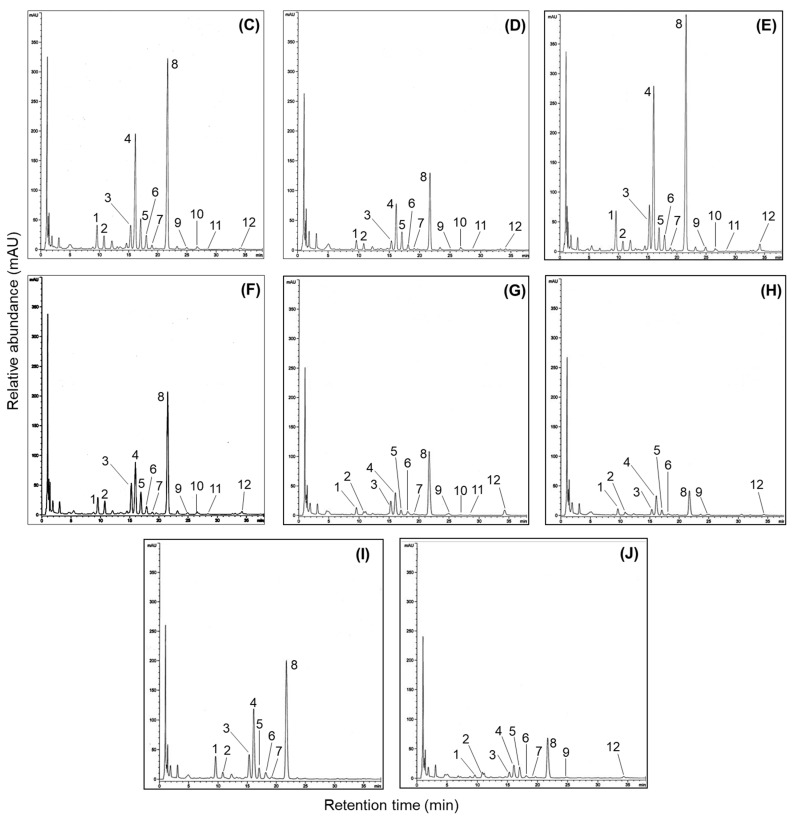
Representative base peak chromatogram and mass fragmentation patterns regarding isoflavone derivatives in the 50% methanol extract of soybean (cv. Sorog) obtained by UPLC-Q-TOF-MS in positive ion mode, and comparison of HPLC chromatograms for 12 isoflavone derivatives in the 50% methanol extracts of soybean cultivars with the highest and the lowest isoflavones in four usage categories. (**A**) UPLC-Q-TOF-MS chromatogram using positive ion mode. Peak 1: daidzin; peak 2: glycitin; peak 3: genistin; peak 4: malonyldaidzin; peak 5: malonylglycitin; peak 6: acetyldaidzin; peak 7: acetylglycitin; peak 8: maloylgenistin; peak 9: daidzein; peak 10: glycitein; peak 11: acetylgenistin; peak 12: genistein. (**B**) Mass fragmentation patterns of isoflavone compositions; (**C**) Shinpaldal 2; (**D**) Daewon; (**E**) Sorog; (**F**) Bukwang; (**G**) Hwaeomput; (**H**) Hwasonput; (**I**) Cheongdu 1; and (**J**) Seonheuk.

Numerous researchers agree that UPLC combined with mass spectrometry is one of the best methods for analyzing the molecular weights of metabolite profiles in natural and agricultural sources [15,17,22]. This approach provides valuable information for metabolite characterization by assigning individual peaks, as the products are generated from the fragmentation of a selected precursor ion [9,25,37]. Therefore, we utilized the mass values obtained from UPLC-ESI-TOF-MS/MS analysis to characterize metabolite patterns, including isoflavone derivatives, in the soybeans. In earlier studies, many people identified various metabolites such as soyasaponins, flavonoids, isoflavones, fatty acids, and lipids in different parts of the soybean plant (seed coat, embryo, seed, cotyledon, and root) [19,25,28,33]. As shown in Figure 4A, the representative BPC chromatogram achieved complete separation of diverse metabolites, including both major and minor peaks, within 40 min. Although the 50% methanol extracts of soybeans contain numerous metabolites, this research focused solely on verifying the presence of isoflavones due to their important contributions to human health, the development of new soybean cultivars, and their functional and pharmaceutical properties. Based on these points, we analyzed the primary constituents, specifically isoflavone derivatives, using UPLC-TOF-MS/MS and evaluated their antioxidant and enzyme inhibition properties. Since isoflavones are considered key metabolites, their presence in soybean extracts was clearly identified using UPLC-TOF-MS/MS analysis. The elucidated isoflavones were presented in full-scan positive ion mode (Figure 4A). The UPLC-ESI-TOF-MS and MS^2^ spectrums of peak 1 (*t_R_* = 13.3 min; mass error: 4.55 ppm) showed the molecular ion [M + H]^+^ at *m*/*z* 417.1204, with the fragment ion observed at *m*/*z* 255.0679. This fragment ion (*m*/*z* 255.0679) may be formed by the loss of a glucose group (162 amu) [30,34]. Based on the mass data and previous literature, peak 1 was assumed to be daidzin [34,37]. Peaks 4 (*t_R_* = 17.0 min; mass error: 4.17 ppm) and 6 *t_R_* = 18.3 min; mass error: 4.35 ppm) displayed molecular ion signals ([M + H]^+^) at *m*/*z* 503.1198 and *m*/*z* 459.1308, with characteristic fragment ions at *m*/*z* 255.0676 and 255.0650. These ions were attributed to daidzein aglycone, resulting from the loss of *m*/*z* 248 and *m*/*z* 204 fragments, respectively. In other words, the fragment ions corresponded to the cleavage of 6″-*O*-malonyl glucoside and 6″-*O*-aetyl glucoside groups [37]. Furthermore, the fragmentation ions (peak 4: daidzein + 248; peak 6: daidzein + 204) of two peaks were consistent with peak 1. In accordance with these ions and earlier evidence, two peaks were confirmed as malonyldaidzin (4) and acetyldaidzin (6). The full mass scan of peak 9 (*t_R_* = 19.3 min; mass error: 9.80 ppm) revealed an identical molecular ion consistent with the fragmentation patterns of peaks 1 (*m*/*z* 255.0679), 4 (*m*/*z* 255.0676), and 6 (*m*/*z* 255.0677). Thus, this peak was tentatively identified as daidzein (9) [4,30,34]. The product ion mass spectrum of peak 2 (*t_R_* = 14.2 min; mass error: 4.47 ppm) showed a molecular ion [M + H]^+^ at *m*/*z* 447.1306 and a fragment ion at *m*/*z* 285.0783, resulting from the loss of the 6″-*O*-glucoside moiety (*m*/*z* 162), as supported by prior studies [30,34]. According to the described ions, this peak was identified as glycitin (2), with the formula C_22_H_22_O_10_. Peak 5 (*t_R_* = 17.4 min; mass error: 3.37 ppm) exhibited a molecular ion [M + H]^+^ at *m*/*z* 533.1304, with a fragment ion at *m*/*z* 285.0781, resulting from a loss of 248 amu. This fragmentation (*m*/*z* 285.0781) was generated by the loss of glucose (162 amu) and malonyl (86 amu) residues and was characterized as a glycitein skeleton (−248 amu) [37]. As a result, peak 5 was tentatively elucidated as malonylglycitin (C_25_H_24_O_13_) [37]. The UPLC-ESI-TOF-MS spectrum of peak 7 (*t_R_* = 18.8 min; mass error: 4.90 ppm) in positive ion mode revealed a molecular ion at *m*/*z* 489.1415, with minor fragment ion at *m*/*z* 285.1220. This fragment ion was assigned as glycitein aglycone, with the loss of an acetylglucoside group (acetyl + glucose: 43 amu + 162 amu), as previously reported [37]. Therefore, peak 7 was tentatively assigned as acetylglycitin of the formular C_24_H_24_O_11_. Subsequently, peak 10 (*t_R_* = 20.1 min; mass error: 7.36 ppm) displayed a precursor ion [M + H]^+^ at *m*/*z* 285.0779, consistent with glycitein-related peaks 2, 5, and 7. For this evidence, peak 10 was assumed to be glycitein (10) of the C_16_H_12_O_6_ structure [4,34]. The remaining isoflavone derivatives, peaks 3, 8, 11, and 12, were identified as genistein compositions based on their characteristic fragmentation patterns. Peak 3 (*t_R_* = 15.8 min; mass error: 4.61 ppm) exhibited a molecular ion at *m*/*z* 433.1149 and a fragment ion at *m*/*z* 271.0626, indicating the loss of a glucose group (*m*/*z* 162) and confirming it as genistin (C_21_H_20_O_10_) [37]. Peak 8 (*t_R_* = 19.1 min; mass error: 3.85 ppm) was observed as a parent ion [M + H]^+^ at *m*/*z* 519.1153 with a significant fragment ion at *m*/*z* 271.0629 ([M + H]^+^ −248 amu). This ion was produced by the sequential loss of a malonyl group (86 amu) and a glucose moiety (162 amu). Additionally, the fragment ion at *m*/*z* 271.0629 was consistent with the fragmentation pattern (*m*/*z* 271.0626) observed in peak 3. By comparing the described ions and the previous data, this peak was identified as malonylgenistin (C_24_H_22_O_13_) [37]. The MS date of peak 11 (*t_R_* = 20.7 min; mass error: 4.84 ppm) revealed a molecular ion at *m*/*z* 475.1258 and a fragment at *m*/*z* 271.0631, showing a similar fragmentation pattern to peaks 3 and 8. The above evidence suggests that peak 11 contains a genistein skeleton, formed by the losses of protonated glucose (162 amu) and acetyl (42 amu) groups. Therefore, this peak was confirmed as acetylgenistin (C_23_H_22_O_11_) [37]. Peak 12 (*t_R_* = 22.8 min; mass error: 9.22 ppm), including molecular ion ([M + H]^+^) at *m*/*z* 271.0626, was elucidated as genistein (C_15_H_10_O_5_) [4,30,37] by examining its fragmentation ions in relation to peaks 3 (*m*/*z* 271.0626), 8 (*m*/*z* 271.0629), and 11 (*m*/*z* 271.0631) (Table S2). Although earlier studies have demonstrated the presence of various metabolites in soybeans, the present research exclusively identified isoflavones (Figure 4B) from several peaks in the 50% methanol extract (Figure 4A) using UPLC-ESI-TOF-MS analysis. This study emphasizes the importance of isoflavone profiling, which is well-recognized for its functional and nutritional contributions to soybean composition. To the best of our knowledge, limited information is available regarding the molecular weight based on the isoflavone distribution in the four soybean types using LC-MS analysis.

### 3.6. Distribution of Isoflavone Contents in Four Categories Based on the Applications of Various Cultivated Soybeans

Our study was structured to explore the isoflavone derivatives in soybean cultivars through usage classifications. Representative HPLC chromatograms showing the isoflavone components in the soybeans are presented in Figure 4C, with each peak detected within 35 min at 254 nm. The 12 isoflavones were identified by referencing previous data and the retention times of standard materials. Their retention times are listed as follows: peak 1 (Din, *t_R_* = 9.6 min), peak 2 (Gly, *t_R_* = 10.7 min), peak 3 (Gin, *t_R_* = 15.3 min), peak 4 (MDin, *t_R_* = 16.1 min), peak 5 (MGly, *t_R_* = 16.9 min), peak 6 (AcDin, *t_R_* = 18.0 min), peak 7 (AcGly, *t_R_* = 19.1 min), peak 8 (MGin, *t_R_* = 21.5 min), peak 9 (Dein, *t_R_* = 24.9 min), peak 10 (Glein, *t_R_* = 26.7 min), peak 11 (AcGin, *t_R_* = 28.4 min), and peak 12 (Gein, *t_R_* = 34.2 min). As presented in Table 1, the individual and total isoflavones differed significantly in 41 soybean cultivars; specifically, total isoflavones in the four usage-based categories exhibited a wide range between cultivars: 1217.0–2825.4 μg/g (bean paste), 2009.6–3722.4 μg/g (bean sprout), 635.3–1102.3 μg/g (vegetable), 692.9–2205.5 μg/g (cooked-with-rice). The average isoflavone contents also exhibited remarkable differences in cultivars and usage categories. The highest average content was found in bean sprout soybeans (2780.6 μg/g), whereas the lowest isoflavone was detected in vegetable soybeans (883.2 μg/g). The remaining categories are displayed in the following order: bean paste (1837.8 μg/g) > cooked-with-rice (1448.2 μg/g) (Table 1). In all the cultivars, the most abundant average group was malonylglucoside with 1335.3 μg/g, representing approximately 76.8% of the total isoflavones (1737.6 μg/g), followed by glucoside (20.6%, 358.6 μg/g), acetylglucoside (1.8%, 32.1 μg/g), and aglycone isoflavone (0.07%, 11.6 μg/g) (Table 1). At the individual isoflavone level, malonylgenistin had the predominant average content (743.4 μg/g), and the contents of other malonyl derivatives were observed in increasing order: malonyldaidzin (378.9 μg/g) > malonylglycitin (213.0 μg/g). The second main group, isoflavone glucosides, exhibited average amounts in the following order: daidzin (131.1 μg/g) > genistin (119.8 μg/g) > glycitin (107.7 μg/g). The average contents of acetylglucoside and aglycone types accumulated in the following sequence: acetyldaidzin (23.8 μg/g) > acetylglycitin (7.1 μg/g) > daidzein (5.5 μg/g) > genistein (4.5 μg/g) > glycitein (1.6 μg/g) > acetylgenistin (1.2 μg/g) (Table 1). These results showed distinct patterns of soybean isoflavones compared to previous research findings [25,28,33]. In the 11 soybean cultivars used for bean paste production, total isoflavone contents ranged from 1217.0 to 2825.4 μg/g. The highest isoflavone was observed in Shinpaldal 2 with 2825.4 μg/g (Figure 4C), and the Samnam cultivar made up the second major content (2506.5 μg/g). Moreover, the lowest content was detected in the Daewon cultivar (1217.0 μg/g) (Figure 4D). Other cultivars were presented in the sequence Malli (2183.0 μg/g) > Dankyeong (1994.8 μg/g) > Jinpum 2 (1969.6 μg/g) > Pokwang (1809.7 μg/g) > Daehwang (1624.9 μg/g), with remaining cultivars showing < 1500 μg/g (Table 1). In the bean sprout soybeans, all the cultivars exhibited high isoflavone concentrations exceeding 2000 μg/g, and considerable differences were detected in individual and total isoflavone contents when compared with those of other soybean categories, with a range of 2009.6–3722.7 μg/g. The absolutely dominant content was presented in Sorog with 3722.7 μg/g (Figure 4E), and other cultivars (>2500 μg/g) were ranked in order by the increase rates as follows: Sowon (3389.4 μg/g) > Sojin (3026.6 μg/g) > Eunha (2870.1 μg/g) > Dagi (2781.7 μg/g) > Soho (2777.2 μg/g) > Iksannamul (2757.4 μg/g) > Myeongjunamul (2631.0 μg/g). The lowest amounts are displayed by Bukwang (2009.6 μg/g) (Figure 4F). This category was also increased in the order malonylglucoside > glucoside > acetylglucoside > aglycone groups, as reported in other usage classifications; specifically, the malonylgenistin content (average 1159.7 μg/g) has remarkable influences on total isoflavone amounts (average 2780.6 μg/g). Another usage category, vegetable soybeans, exhibited considerably low isoflavone levels, with a range of 635.3–1102.3 μg/g, compared to the other usage classifications. In particular, the average isoflavone content (883.2 μg/g) was three times less than that of the bean sprout category (2780.6 μg/g). Also, most vegetable soybean cultivars exhibited slight variations in isoflavone contents, with Hwaeomput (1102.3 μg/g) (Figure 4G) > Danmi (1030.8 μg/g) > Shillog (931.5 μg/g) > Seokryangput (912.6 μg/g) > other cultivars (757.7–856.7 μg/g), and the Hwasonput cultivar showed the lowest content of 635.3 μg/g (Figure 4H). Cooked-with-rice soybeans exhibited significant differences with the ranges of 802.9–2205.5 μg/g in 11 cultivars. The Cheongdu 1 displayed abundant isoflavones, accounting for 2205.5 μg/g (Figure 4I), and the second main isoflavones were obtained from Ilpumgeomjeong (2083.1 μg/g), followed by Geomjeong 2 (2009.8 μg/g) > Heugcheong (1761.2 μg/g) > Geomjeongol (1572.5 μg/g), and the remaining cultivars were detected with <1500 μg/g contents. In addition, the lowest distributions were measured in the Seonheuk cultivar (692.9 μg/g) (Figure 4J).

### 3.7. Correlation Analysis of Metabolite Constituents in Four Usage Categories Based on Various Cultivated Soybeans

**Figure 5:**
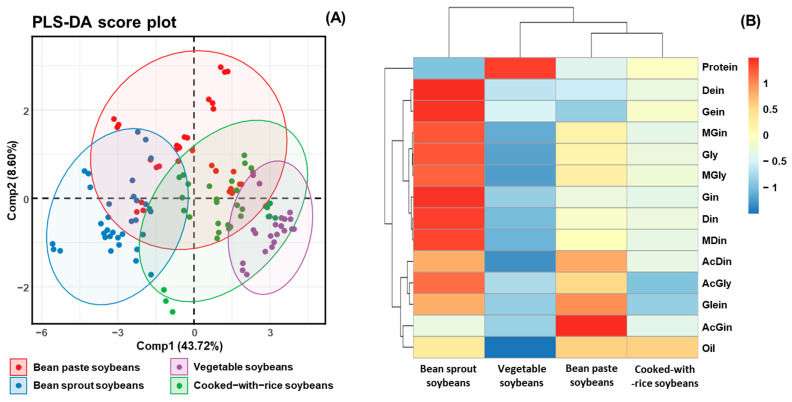
Score plot of PLS-DA and heatmap analysis of metabolites (protein, oil, and isoflavone) for 41 soybean cultivars in four usage categories. (**A**) Score plot of PLS-DA of four soybean usage categories; and (**B**) hierarchical clustering and heatmap analysis to investigate the variable metabolites’ relationships, either positive (red) or negative (blue).

To elucidate the interrelationships among metabolite constituents in soybeans, PLS-DA and hierarchical clustering were conducted using metabolite profiling data from 41 cultivars, which were classified into four usage categories: bean sprout soybeans, vegetable soybeans, bean paste soybeans, and cooked-with-rice soybeans (Figure 5A,B) [38]. PLS-DA was employed to examine the interrelationship among metabolite constituents, including isoflavone, protein, and oil in different soybean usage classifications (Figure 5A). A heatmap was generated based on z-scores to quantitatively evaluate the metabolites, illustrating the hierarchical clustering of metabolites within each sample. The heatmap visually represents the positive (red) and negative (blue) associations among metabolites, providing insights into their interdependencies (Figure 5B) [25,36]. The results from the PLS-DA revealed that the component (Comp) 1 and Comp2 accounted for 43.72% and 8.60% of the total variance, respectively, leading to a cumulative variance of approximately 54%. This suggests that Comp1 and Comp2 together capture a substantial portion of the variability in the dataset and provide meaningful insights into the major trends and groupings. Nevertheless, this cumulative variance does not meet the commonly accepted threshold of 75% for sufficient data representation in PLS-DA. Notably, it was not until Comp6 that the cumulative variance reached 76.31%, indicating that a higher number of components is required to adequately explain the underlying data structure (Table S3). In the two-dimensional scatter plot of factor loadings (Figure 5A), the X-axis represents Comp1, while the Y-axis reflects Comp2. The 95% confidence ellipses demonstrated that vegetable and bean sprout soybeans formed distinct clusters, underscoring the distinct differences in their metabolite profiles. In contrast, cooked-with-rice and bean paste soybeans clustered closely together, suggesting a high degree of similarity in their metabolite compositions. Among the 41 cultivars analyzed for protein, oil, and isoflavone contents, bean sprout soybeans exhibited higher concentrations of these metabolites compared to the other usage categories. In contrast, cooked-with-rice and bean paste soybeans displayed similar metabolite profiles, forming a closely related group. Vegetable-type soybeans, which contained the lowest levels of these metabolites, were positioned separately as a distinct group. Notably, except for bean paste soybeans, all other clusters exhibited a wide range along Comp2, indicating that glycoside isoflavone contents significantly influenced the clustering distribution (Figure 5A). A heatmap, including a dendrogram, was constructed to further visualize the distribution of protein, oil, and isoflavone contents in various cultivated soybeans (Figure 5B) [25,36]. The results corroborated the PLS-DA findings, demonstrating clear group separations between bean sprout and vegetable soybeans. Moreover, the correlation coefficients for protein and oil contents in each usage category did not show similarities to the correlation coefficients of isoflavones. It can be concluded that only the 12 isoflavone compositions significantly contributed to the soybean clustering patterns. Additionally, clustering was distinguished based on high and low levels of protein, oil, and isoflavone contents.

## 4. Discussion

Several researchers have extensively employed in vitro techniques, including radical scavenging, to screen for antioxidants in foods and natural plants, owing to their simplicity, reliability, and reproducibility when analyzed via spectrophotometry [21,28,35]. The scavenging effects against two radicals varied significantly in the cultivars and usage categories. These findings imply that the isoflavone concentrations present in soybean extracts may significantly contribute to their antioxidant properties through enhanced radical scavenging abilities [21,24,28,34]. Moreover, the unidentified peaks (Figure 4A) observed in the 50% methanol extracts could serve as potential contributors to the two radical scavenging characteristics, as indicated by prior studies [30,39]. Interestingly, all sample extracts demonstrated greater ABTS radical scavenging capacities compared to the DPPH radical results. This phenomenon may be attributed to the fact that the DPPH radical is primarily associated with the hydrogen-donating antioxidant abilities of isoflavones and other metabolites found in soybeans, whereas the inhibition of the ABTS radical may involve both chain-breaking and hydrogen-donating antioxidant mechanisms [9,19,40]. As mentioned above, the bean sprout soybeans emerge as promising candidates for the development of improved cultivars and functional foods owing to their high isoflavone contents and strong antioxidant effects. To further elucidate these findings, Figure 1A–H presents a comparative analysis of soybean cultivars, highlighting those with the highest radical scavenging effects based on their respective applications. Therefore, the DPPH radical scavenging effects are likely positively correlated with the accumulation and profiles of isoflavones in soybeans [28,34,35]. The current data are consistent with previous studies reporting similar antioxidant patterns in isoflavone extracts from various soybean cultivars [32,39]. Other metabolites in soybean extracts may also be responsible for the major portion of scavenging properties against this radical [9,25,30]. In other words, the four soybean cultivars, categorized by their applications, demonstrated higher scavenging capacities, showing approximately 20–30% higher values than those observed with the DPPH assay at each concentration (Figure 1E–H). These observations were in agreement with findings reported in previous literature [28,35,40]. This evidence suggests that Sorog and Shinpaldal 2 could be considered as effective natural antioxidant sources for functional and nutraceutical applications. Specifically, cultivars intended for bean sprout applications may be valuable as alternative dietary supplements in human foods, owing to their potent radical scavenging activities and high isoflavone contents.

It is well established that phenolic metabolites and their contents are recognized as agents that help protect against DNA damage [19,36]. Numerous recent studies have demonstrated that natural plants, such as foods, crops, and vegetables, possess outstanding functional properties in preventing DNA damage due to their antioxidant effects [23,41]. However, there are still limited reports on the DNA protection effects of recombinant extracts in soybean cultivars categorized by usage, which are needed to better understand their antioxidant properties. These findings suggest that isoflavones and other antioxidant-related metabolites in soybean extracts are likely to contribute significantly to DNA protection, as reported in previous studies [19,27]. We have confidence that the 50% methanol extract of the Sorog cultivar may serve as an excellent candidate for developing health-beneficial agents and protecting against oxidative DNA damage, adding functional value to soybeans. Moreover, bean sprout soybeans may be particularly important for human health due to their higher DNA protection and radical scavenging capacities compared to other groups. To our knowledge, this is the first study to provide a comparative evaluation of DNA protection and radical scavenging properties in various soybean cultivars categorized by their intended applications.

Many researchers are exploring new tyrosinase and α-glucosidase inhibitors derived from natural and edible sources due to the adverse side effects associated with synthetic drugs, seeking materials that offer enhanced safety characteristics [9,10,11,14,23]. Due to the adverse side effects associated with synthetic drugs, many researchers are exploring novel tyrosinase and α-glucosidase inhibitors derived from natural and edible sources, seeking materials with enhanced safety profiles [19,23,34]. In particular, recent studies have demonstrated that isoflavones and phenolic metabolites in soybeans are primarily responsible for their enzyme inhibitory properties [9,13,24,34]. These compounds exert enzyme inhibitory effects primarily through specific binding and redox mechanisms, which are strongly influenced by their structural features. For example, variations in the hydroxylation patterns of major soy isoflavones such as genistein and daidzein affect their affinity for enzyme active sites and redox activity, thereby modulating enzymatic function. This suggests that the structural diversity of isoflavones plays a key role in their biological efficacy [19,34,42,43]. However, research evaluating enzyme inhibition in diverse soybean cultivars based on their utilization remains limited. In other words, bean sprout soybeans exhibited remarkably higher average tyrosinase and α-glucosidase inhibition compared to other applications, while vegetable soybeans showed the lowest inhibitory effects (Figure 3). These investigations may be attributed to differences in isoflavone contents among each soybean, as noted in previous studies [13,34]. Furthermore, other phenolic metabolites, including phenolic acid, pterocarpan, and flavonoid derivatives in soybean extracts, may contribute to the primary mechanism of two enzyme inhibitions [9,19]. Notably, our findings are consistent with the order of the above reported data on isoflavone contents and antioxidant abilities. In addition, the enzyme inhibition results observed in bean sprout soybeans (tyrosinase: ascorbic acid, 49.17 mg/g; α-glucosidase: acarbose, 44.70 mg/g at 500 μg/mL after 3 min reaction; Table S1) suggest their potential as valuable sources for human health and the development of new cultivars. The present findings are consistent with previous research showing that higher isoflavones in soybeans are associated with increased tyrosinase inhibition [13,34]. Apart from isoflavones, other phenolic metabolites present in the extracts may contribute significantly to tyrosinase inhibition by forming hydrogen bonds with the enzyme’s active site, leading to conformational changes and increased steric hindrance [11,14]. Even though most soybean extracts, except for the Sorog cultivar, displayed lower inhibitions than the positive control, our results can provide valuable information on effective anti-diabetic sources for the development of commercial and functional foods. As observed in the tyrosinase experiment results, our data may be influenced by the isoflavone contents and the presence of trace phenolic metabolites (phenolic acids, flavonoids, etc.) found in soybean extracts [13,19,43]. These investigations are consistent with reported results on the patterns of isoflavone contents and antioxidant properties. Overall, our research suggests that the Sorog cultivar of bean sprout soybeans may be recommended as a potent source for developing health-promoting agents due to its strong tyrosinase and α-glucosidase inhibition, as well as high isoflavone contents and antioxidant properties.

Soybeans are well known for their potential in producing high-protein products for human consumption [18] and their significant role in biofuel production [16]. These applications are considerably influenced by the protein and oil contents. Therefore, improving overall management and prediction of soybean protein and oil concentrations at the field level is essential for maximizing added value in soybean production, enhancing quality, and developing superior soybean cultivars. To the best of our knowledge, there is no existing information that compares protein and oil contents based on soybean usages in diverse cultivars. Generally, soybeans contain an average of 36–42% protein and 18–20% oil [18,25,31,35]. Although our data were similar to the previous literature [18,19], the protein content of soybeans appears to be less affected by environmental changes, including temperature, year, growth state, soil nutrition, and light [18]. However, this constituent may be partially influenced by genetic differences, as has been demonstrated in vegetable soybeans [33]. Therefore, vegetable soybean cultivars are of great interest to food manufacturers and researchers because of their high protein accumulation. In addition, protein may play a role as important as isoflavone in determining quality and functionality through soybean utilization. Particularly, vegetable soybeans may be recommended as a potential cultivar due to their high protein content. Although soybean oil content is commonly significantly influenced by diverse factors, including environmental conditions and food processing [16,19], the present research did not reveal notable differences in cultivars and genetics. Based on the above findings, oil content may not be a crucial factor in assessing the soybean properties. Overall, the oil content of soybeans does not appear to have a strong positive correlation with cultivars, usage categories, or their internal relationships. This study is the first to compare protein and oil contents in relation to different usage classifications from various Korean soybean cultivars.

Several researchers have demonstrated comparisons and variations of isoflavone and other metabolite accumulations in soybeans and their products under environmental parameters [9,21,28,35]. However, as far as we are aware, investigations regarding changes in isoflavone composition in diverse soybean cultivars categorized by their intended usage have not yet been extensively conducted. Therefore, in this study, we separated single-variety soybeans into four cooking uses (bean paste, bean sprouts, vegetable, and cooked-with-rice) and rigorously verified whether the differences in intrinsic physiological activity could be predicted. Previous reports for phenolic metabolites of other crops suggest that the isoflavone contents in each cultivar may be markedly affected by genetic diversities [6,7,11]. Our results were consistent with earlier studies showing that secondary metabolites such as isoflavones and anthocyanins differ considerably in black soybeans during crop years [19]. We suggest that isoflavone accumulation is closely related to the levels of chalcone synthase and isoflavone synthase in the phenylpropanoid pathway, along with cellular division and biosynthesis, as well as cultivation techniques and environmental conditions during the growth of the soybean plant [1,29,30,34]. Our results are similar to the previous literature that the predominant individual isoflavone observed in soybeans was malonylgenistin [25,28,34]. Although some researchers have reported malonyldaizin as the most abundant component, this phenomenon appeared to be influenced by various factors, such as genetics, growth conditions, and other environmental elements [9,19,33]. The substantial differences observed in the cultivars were largely influenced by the high variability of malonylgenistin, which ranged from 237.0 to 1105.8 μg/g compared to other isoflavone derivatives [28]. Our findings are consistent with previous reports that the levels of secondary metabolites in natural plants and crops can fluctuate owing to diverse parameters [1,4,6,24,28,36,37]. Even though several studies have evaluated soybean isoflavones, the current work uniquely quantified and compared the individual and total contents in different usage-based categories for the first time, using various cultivars. As evidenced by the above results, we can conclude that bean sprout soybeans have significant potential as valuable sources for developing improved cultivars and functional foods due to their high isoflavone ratio. In particular, three cultivars (Sorog, Sowon, and Sojin), with isoflavone concentrations greater than 3000 μg/g, are suitable for use in the manufacturing of nutraceutical products and health foods. We believe that isoflavone content is a critical parameter in evaluating soybean quality.

**Figure 6:**
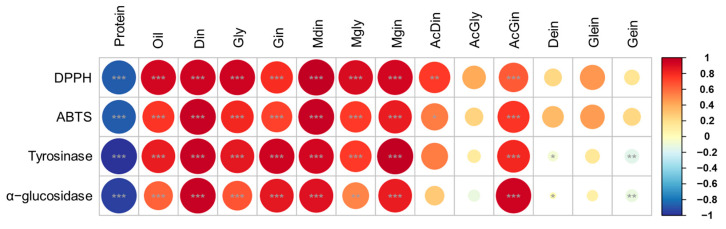
Pearson correlations between metabolites (protein, oil, and isoflavone) and biological activities for soybean cultivars in four usage categories. Red–blue colors represent positive and negative correlations. *, *p* < 0.05; ** *p* < 0.01; and *** *p* < 0.001.

This analysis is critical for understanding how the metabolites vary with respect to the intended applications of soybeans, such as food processing, oil extraction, and direct consumption [9,28,36]. For instance, daidzin, genistin, and malonyldaidzin formed one cluster, while glycitin, malonylglycitin, and malonylgenistin formed another. The remaining isoflavone compositions clustered together with oil constituents. Overall, bean paste and cooked-with-rice soybeans, which exhibited similar patterns in protein and isoflavone contents, showed correlation coefficients close to 0 and formed a single cluster (Figure 5B). In contrast, bean sprout and vegetable soybeans, characterized by significantly different compositions, displayed correlation coefficients close to 1 and −1, respectively, indicating a lack of correlation between these two usage categories (Figure 5B). The correlation analysis of four categories of soybean extracts—namely, protein, oil, isoflavone derivatives, and functional activity—indicated that the oil content and malonyl and β-glycoside isoflavones (including malonyldaidzin, malonylglycitin, malonylgenistin, daidzin, glycitin, and genistin) exhibited very strong correlations (r > 0.95) with radical scavenging and enzyme inhibition activities (Figure 6). In contrast, the protein fraction, acetylated isoflavones, and aglycones demonstrate minimal correlations. These findings align with previous research which suggests that malonyl bonds enhance antioxidant potency by improving electron delocalization and solubility [19,20]. This structure–activity relationship highlights that choosing soybean cultivars characterized by elevated malonyl isoflavone contents can optimize antioxidant efficacy while producing extracts that are effective for inhibiting tyrosinase and α-glucosidase. This emphasizes the essential importance of cultivar selection and metabolite profiling in the advancement of health-promoting and functional soybean food products [9,37].

## 5. Conclusions

This research is the first to provide comprehensive insights into the comparisons of biofunctional metabolites and biological properties from various cultivated soybeans categorized according to their intended usage for the development of beneficial food ingredients and cultivars. Twelve isoflavone derivatives in the 50% methanol extract of soybean were verified using UPLC-Q-TOF-MS/MS, and their accumulations were evaluated by HPLC analysis. The isoflavone contents displayed considerable differences between cultivars and usage classifications, with the total average contents exhibiting the following order: bean sprout (2780.6 μg/g) > bean paste (1837.8 μg/g) > cooked-with-rice 1448.2 μg/g) > vegetable (883.2 μg/g). In addition, the malonylglucoside type emerged as the predominant average content, with 1335.3 μg/g in all cultivars, representing about 76.8% of the total isoflavones (1737.6 μg/g), and malonylgenistin showed the highest content, averaging 743.4 μg/g (42.3%). Other essential constituents of soybeans, such as protein and oil, exhibited no substantial differences, with ranges of 40.0–44.9% and 18.6–20.5%, respectively. Specifically, vegetable soybeans exhibited the highest average protein content (44.9%) and the lowest average oil content (18.6%). The antioxidant properties, such as radical scavenging and DNA protection ratios, as well as tyrosinase and α-glucosidase inhibitions, showed significant differences in a concentration-dependent manner from cultivars and usage categories, with their capacities observed in the same order as the isoflavone distribution patterns. Among various assays, the ABTS radical scavenging activities displayed the highest beneficial properties (>80% at 500 μg/mL) compared to other biological tests. These findings may provide valuable evidence that the isoflavone accumulations in soybeans are attributed to the antioxidant and enzyme inhibition ratios. Interestingly, soybean cultivars used for bean sprout applications may serve as potential sources for the development of human nutraceutical agents. In particular, the Sorog cultivar within the bean sprout group can be considered as an alternative natural candidate for the development of new soybean types and health-oriented soy-based food products. Future research is needed to explore the health benefits and the development of commercial products related to bean sprout classification.

## Figures and Tables

**Table 1:** Comparisons of isoflavone contents in four categories based on Korean soybean usage.

Category	Cultivar	Isoflavone contents (μg/g) ^1^												
		Glucoside			Malonylglucoside			Acetylglucoside			Aglycone			Total
		Din (1)	Gly (2)	Gin (3)	MDin (4)	MGly (5)	MGin (8)	AcDin (6)	AcGly (7)	AcGin (11)	Dein (9)	Glein (10)	Gein (12)	
Bean paste soybeans	Shinpaldal 2	167.0 ± 10.4 ^ef^	184.5 ± 10.6 ^e^	113.1 ± 3.7 ^g^	672.3 ± 31.9 ^e^	419.7 ± 19.5 ^b^	1172.5 ± 40.8 ^de^	54.0 ± 2.5 ^b^	18.7 ± 1.4 ^e^	tr	7.4 ± 0.7 ^h^	12.1 ± 0.9 ^d^	4.1 ± 0.3 ^l^	2817.9 ± 40.3 ^d^
	Taekwang	85.8 ± 4.3 ^tu^	89.4 ± 5.1 ^m^	33.6 ± 1.9 ^qr^	265.6 ± 13.4 ^no^	240.4 ± 9.5 ^i^	469.6 ± 15.1 ^pq^	27.4 ± 0.9 ^j^	11.8 ± 0.3 ^k^	tr	tr	10.5 ± 0.2 ^e^	2.7 ± 0.1 ^m^	1252.0 ± 24.0 ^r^
	Samnam	186.9 ± 13.6 ^de^	208.2 ± 11.7 ^ab^	170.1 ± 9.3 ^e^	527.3 ± 30.2 ^g^	322.0 ± 14.1 ^ef^	1040.4 ± 46.3 ^fg^	25.3 ± 0.7 ^k^	17.1 ± 0.2 ^f^	nd	9.2 ± 0.1 ^f^	tr	tr	2519.5 ± 46.3 ^g^
	Pokwang	128.7 ± 4.5 ^kl^	139.3 ± 3.9 ^j^	74.5 ± 2.7 ^jk^	387.2 ± 17.6 ^j^	246.0 ± 8.0 ^hi^	785.0 ± 31.5 ^l^	33.9 ± 1.3 ^e^	14.1 ± 0.3 ^i^	1.0 ± 0.0 ^f^	tr	tr	tr	1807.7 ± 23.8 ^n^
	Dankyeong	151.7 ± 10.9 ^gh^	97.0 ± 6.3 ^m^	98.3 ± 5.7 ^h^	513.0 ± 24.1 ^g^	210.6 ± 10.3 ^j^	874.1 ± 28.4 ^k^	33.8 ± 1.6 ^e^	14.1 ± 0.2 ^i^	nd	2.2 ± 0.1 ^n^	tr	tr	2002.1 ± 9.8 ^kl^
	Jinpum 2	114.4 ± 6.7 ^no^	151.9 ± 12.7 ^gh^	68.6 ± 2.8 ^mn^	448.5 ± 20.2 ^h^	411.7 ± 17.9 ^b^	730.2 ± 26.5 ^l^	30.2 ± 1.1 ^h^	13.9 ± 0.1 ^i^	tr	0.2 ± 0.0 ^q^	tr	tr	1973.9 ± 13.5 ^l^
	Daewon	104.6 ± 5.3 ^op^	148.8 ± 5.4 ^hi^	21.1 ± 1.0 ^s^	170.7 ± 9.5 ^rs^	109.5 ± 3.8 ^no^	578.2 ± 17.9 ^no^	35.5 ± 0.8 ^d^	tr	35.2 ± 0.6 ^a^	4.7 ± 0.1 ^j^	tr	8.7 ± 0.3 ^g^	1225.5 ± 19.2 ^r^
	Jangwon	102.0 ± 4.7 ^qr^	139.4 ± 4.5 ^j^	86.0 ± 3.2 ^ij^	232.5 ± 11.8 ^pq^	217.1 ± 7.0 ^j^	638.1 ± 18.3 ^n^	34.2 ± 1.3 ^de^	15.5 ± 0.7 ^g^	0.2 ± 0.0 ^i^	1.4 ± 0.0 ^o^	tr	0.2 ± 0.0 ^u^	1451.2 ± 29.6 ^p^
	Daehwang	97.2 ± 4.1 ^qr^	54.4 ± 3.6 ^op^	85.4 ± 4.0 ^ij^	315.3 ± 15.4 ^lm^	122.2 ± 4.7 ^n^	900.7 ± 39.7 ^jk^	32.5 ± 1.4 ^f^	14.4 ± 0.5 ^hi^	tr	2.8 ± 0.1 ^l^	tr	tr	1636.4 ± 36.6 ^o^
	Daemang	104.3 ± 6.2 ^op^	45.7 ± 1.9 ^pq^	82.7 ± 4.3 ^ij^	305.8 ± 12.7 ^lm^	81.8 ± 2.1 ^pq^	726.1 ± 29.6 ^lm^	33.6 ± 1.7 ^e^	tr	tr	2.1 ± 0.3 ^n^	tr	nd	1368.6 ± 16.3 ^q^
	Malli	164.3 ± 9.3 ^fg^	152.4 ± 5.0 ^gh^	186.8 ± 8.8 ^cd^	418.8 ± 22.9 ^hi^	210.4 ± 7.3 ^j^	995.4 ± 35.0 ^gh^	26.9 ± 1.2 ^jk^	tr	2.8 ± 0.5 ^c^	8.3 ± 0.5 ^g^	15.8 ± 0.8 ^a^	1.1 ± 0.1 ^r^	2177.6 ± 38.3 ^ij^
Bean sprout soybeans	Eunha	215.0 ± 12.2 ^c^	198.8 ± 9.4 ^cd^	194.5 ± 12.0 ^c^	680.7 ± 35.1 ^e^	329.6 ± 14.3 ^ef^	1207.0 ± 45.5 ^cd^	33.6 ± 0.9 ^e^	nd	tr	7.4 ± 0.4 ^h^	tr	3.5 ± 0.3 ^n^	2893.8 ± 72.2 ^d^
	Soho	268.6 ± 13.6 ^a^	113.0 ± 4.0 ^kl^	173.8 ± 10.2 ^e^	966.2 ± 41.3 ^b^	243.7 ± 7.9 ^i^	949.9 ± 33.1 ^hi^	26.3 ± 0.6 ^jk^	14.7 ± 0.3 ^h^	tr	16.9 ± 0.7 ^e^	1.8 ± 0.5 ^g^	2.3 ± 0.2 ^o^	2771.2 ± 17.7 ^e^
	Myeongjunamul	192.0 ± 7.3 ^d^	110.2 ± 4.5 ^kl^	249.6 ± 13.7 ^ab^	506.1 ± 25.0 ^g^	181.6 ± 8.1 ^k^	1289.9 ± 37.9 ^c^	30.1 ± 0.8 ^h^	29.5 ± 0.9 ^a^	4.1 ± 0.4 ^b^	21.3 ± 1.0 ^c^	tr	16.6 ± 0.7 ^e^	2602.9 ± 34.1 ^f^
	Iksannamul	233.5 ± 11.4 ^b^	166.3 ± 5.9 ^fg^	178.8 ± 9.5 ^de^	801.6 ± 33.8 ^c^	340.3 ± 12.0 ^e^	1004.1 ± 39.1 ^gh^	29.2 ± 0.5 ^i^	nd	tr	0.9 ± 0.0 ^p^	2.7 ± 0.5 ^f^	tr	2733.8 ± 19.8 ^e^
	Sobaegnamul	120.9 ± 5.6 ^lm^	187.2 ± 9.6 ^de^	187.9 ± 12.4 ^cd^	300.2 ± 16.9 ^mn^	358.9 ± 11.6 ^de^	1059.5 ± 42.9 ^ef^	24.7 ± 0.6 ^kl^	22.1 ± 0.7 ^c^	tr	27.0 ± 1.1 ^b^	0.2 ± 0.0 ^i^	1.7 ± 0.1 ^p^	2286.0 ± 32.9 ^h^
	Bukwang	133.6 ± 5.9 ^jk^	197.6 ± 10.7 ^cd^	150.3 ± 7.1 ^f^	340.7 ± 16.3 ^kl^	341.5 ± 13.4 ^de^	769.9 ± 26.1 ^l^	37.1 ± 0.9 ^c^	11.9 ± 0.3 ^k^	tr	7.0 ± 0.3 ^h^	13.0 ± 0.4 ^c^	7.0 ± 0.3 ^i^	2048.3 ± 19.0 ^k^
	Anpyeong	149.4 ± 7.3 ^hi^	228.9 ± 14.3 ^a^	185.4 ± 9.6 ^cd^	381.8 ± 20.0 ^ij^	337.6 ± 12.7 ^ef^	990.3 ± 33.0 ^gh^	22.9 ± 0.4 ^n^	21.1 ± 0.9 ^d^	2.2 ± 0.5 ^d^	nd	tr	10.8 ± 0.3 ^f^	2332.2 ± 2.8 ^h^
	Dagi	181.5 ± 8.1 ^d^	206.1 ± 12.9 ^bc^	161.2 ± 9.0 ^f^	614.0 ± 27.5 ^f^	388.8 ± 15.2 ^c^	1135.9 ± 34.6 ^ef^	34.5 ± 0.7 ^de^	22.4 ± 0.8 ^c^	1.8 ± 0.5 ^d^	16.1 ± 0.7 ^e^	1.8 ± 0.2 ^g^	17.6 ± 0.2 ^d^	2777.1 ± 44.7 ^e^
	Sorog	255.0 ± 12.6 ^b^	164.9 ± 9.4 ^fg^	236.1 ± 11.3 ^b^	1038.3 ± 35.0 ^a^	366.9 ± 12.3 ^d^	1522.2 ± 49.2 ^b^	63.8 ± 2.0 ^a^	16.9 ± 0.2 ^f^	tr	19.1 ± 0.6 ^d^	13.7 ± 0.3 ^b^	25.8 ± 1.1 ^c^	3746.1 ± 82.4 ^a^
	Sowon	210.0 ± 13.4 ^c^	168.2 ± 7.8 ^fg^	264.6 ± 12.5 ^a^	723.4 ± 30.9 ^de^	319.7 ± 11.9 ^fg^	1669.5 ± 43.9 ^a^	31.5 ± 1.1 ^g^	nd	0.8 ± 0.0 ^g^	1.7 ± 0.2 ^m^	tr	tr	3385.4 ± 67.5 ^b^
	Sojin	212.7 ± 11.0 ^c^	223.5 ± 12.0 ^ab^	181.1 ± 8.6 ^de^	719.2 ± 29.6 ^d^	465.4 ± 13.2 ^a^	1158.4 ± 36.4 ^de^	33.6 ± 1.3 ^e^	19.4 ± 0.6 e	tr	5.5 ± 0.6 ^i^	tr	7.8 ± 0.5 ^h^	3032.2 ± 40.7 ^c^
Vegetable soybeans	Keunol	84.0 ± 3.9 ^uv^	nd ^2^	52.1 ± 2.0 ^op^	172.9 ± 8.3 ^rs^	98.8 ± 3.7 ^no^	438.5 ± 13.6 ^qr^	tr	10.4 ± 0.3 ^l^	nd	nd	tr	tr	853.8 ± 20.7 ^v^
	Seokryangput	94.1 ± 3.5 ^rs^	74.2 ± 2.0 ^n^	59.1 ± 2.9 ^no^	201.2 ± 10.4 ^qr^	113.5 ± 6.3 ^no^	336.7 ± 10.5 ^tu^	33.8 ± 0.8 ^e^	nd	nd	nd	nd	tr	924.2 ± 9.3 ^u^
	Hwaeomput	87.3 ± 2.7 st	70.4 ± 2.4 ^no^	78.8 ± 3.6 ^jk^	184.1 ± 11.7 rs	100.6 ± 5.1 ^no^	518.2 ± 14.0 ^op^	25.7 ± 0.6 ^k^	7.3 ± 0.2 ^m^	tr	9.3 ± 0.5 ^f^	tr	20.6 ± 0.9 ^d^	1107.2 ± 10.7 st
	Hwasonput	72.7 ± 2.3 ^wx^	57.2 ± 1.5 ^op^	26.3 ± 1.4 ^rs^	154.4 ± 6.9 st	97.8 ± 3.0 ^no^	208.2 ± 9.2 ^w^	13.8 ± 0.4 ^m^	nd	nd	3.4 ± 0.3 ^k^	nd	1.5 ± 0.2 ^q^	636.0 ± 12.5 ^z^
	Danmi	112.0 ± 5.4 ^no^	99.4 ± 3.2 ^lm^	72.2 ± 3.1 ^kl^	248.4 ± 13.7 ^p^	69.8 ± 2.5 ^r^	424.6 ± 12.0 ^qr^	tr ^3^	nd	nd	3.2 ± 0.2 ^k^	nd	1.2 ± 0.1 ^r^	1043.9 ± 3.5 ^t^
	Shillog	81.5 ± 3.0 ^vw^	50.5 ± 1.7 ^p^	79.7 ± 3.2 ^ij^	178.9 ± 7.5 ^rs^	80.0 ± 3.9 ^qr^	460.9 ± 11.8 ^pq^	tr	nd	nd	tr	tr	tr	917.0 ± 14.2 ^u^
	Seonnog	58.1 ± 2.1 ^xy^	47.1 ± 1.4 ^p^	52.4 ± 2.3 ^op^	113.9 ± 6.0 ^tu^	63.5 ± 2.1 ^r^	422.7 ± 13.1 ^rs^	tr	nd	nd	tr	nd	tr	762.3 ± 8.1 ^x^
	Dajin	88.0 ± 3.0 st	139.7 ± 3.8 ^ij^	39.5 ± 1.7 ^pq^	174.3 ± 7.0 ^rs^	121.4 ± 6.1 ^mn^	270.4 ± 10.4 ^vw^	tr	nd	nd	5.5 ± 0.2 ^i^	nd	tr	848.6 ± 3.1 ^v^
Cooked-with-rice soybeans	Ilpumgeomjeong	160.0 ± 7.4 ^fg^	31.5 ± 2.0 ^qr^	121.0 ± 4.8 ^g^	510.9 ± 24.6 ^g^	245.7 ± 7.9 ^hi^	919.3 ± 28.4 ^ij^	33.3 ± 0.7 ^e^	nd	2.8 ± 0.4 ^c^	26.6 ± 0.7 ^b^	tr	32.0 ± 1.2 ^b^	2086.9 ± 21.5 ^k^
	Geomjeong 2	143.5 ± 5.5 ^ij^	29.1 ± 1.1 ^r^	151.3 ± 6.3 ^f^	425.5 ± 17.2 ^hi^	154.3 ± 5.8 ^l^	1105.8 ± 36.7 ^ef^	nd	nd	0.3 ± 0.0 ^h^	tr	tr	nd	1995.0 ± 55.5 ^kl^
	Geomjeongol	135.3 ± 6.0 ^jk^	165.4 ± 4.3 ^fg^	60.0 ± 2.4 ^no^	468.2 ± 21.8 ^h^	194.9 ± 6.9 ^jk^	510.1 ± 15.4 ^op^	34.0 ± 0.5 ^e^	nd	nd	2.8 ± 0.2 ^l^	nd	1.8 ± 0.3 ^p^	1551.6 ± 19.2 ^op^
	Geomjeong 1	99.6 ± 4.1 ^pq^	164.0 ± 5.2 ^fg^	50.4 ± 3.1 ^op^	242.4 ± 10.6 ^p^	207.2 ± 7.3 ^j^	374.4 ± 9.8 st	nd	nd	nd	7.3 ± 0.5 ^h^	tr	8.0 ± 0.7 ^h^	1168.3 ± 12.0 ^s^
	Geomjeongsaeol	99.1 ± 4.7 ^qr^	122.0 ± 3.5 ^k^	45.4 ± 2.0 ^pq^	169.1 ± 6.4 ^rs^	96.8 ± 2.9 ^op^	237.0 ± 11.0 ^w^	33.5 ± 0.8 ^e^	nd	tr	tr	nd	tr	807.9 ± 4.5 ^w^
	Jinyul	51.5 ± 2.7 ^y^	95.7 ± 3.7 ^lm^	25.8 ± 1.5 ^rs^	112.0 ± 5.3 ^u^	193.2 ± 7.5 ^jk^	337.7 ± 8.9 ^uv^	nd	nd	1.2 ± 0.1 ^e^	2.7 ± 0.3 ^l^	tr	3.9 ± 0.4 ^l^	824.4 ± 2.2 ^v^
	Heugcheong	154.3 ± 8.1 ^fg^	178.4 ± 8.9 ^ef^	112.0 ± 3.9 ^g^	419.8 ± 20.7 ^h^	199.5 ± 7.0 ^jk^	651.6 ± 15.3 ^mn^	34.1 ± 0.7 ^e^	nd	nd	5.9 ± 0.5 ^i^	tr	5.6 ± 0.5 ^j^	1767.5 ±13.7 ^m^
	Seonheuk	77.8 ± 2.9 ^vw^	98.4 ± 3.3 ^m^	45.3 ± 2.1 ^pq^	89.9 ± 4.1 ^u^	83.9 ± 4.3 ^pq^	288.5 ± 13.6 ^vw^	nd	tr	nd	4.5 ± 0.3 ^j^	nd	4.6 ± 0.4 ^k^	693.2 ± 14.0 ^y^
	Cheongdu 1	109.9 ± 5.0 ^mn^	144.0 ± 4.0 ^j^	153.4 ± 6.7 ^f^	365.5 ± 22.5 ^jk^	299.2 ± 14.9 ^g^	1087.3 ± 30.8 ^ef^	38.1 ± 1.0 ^c^	8.1 ± 0.3 ^n^	nd	nd	nd	nd	2223.1 ±29.3 ^i^
	Geomjeong 3	115.0 ± 4.3 ^mn^	24.4 ± 1.3 ^r^	68.6 ± 3.4 ^lm^	309.9 ± 19.4 ^lm^	267.9 ± 15.4 ^h^	655.6 ± 21.4 ^mn^	35.8 ± 1.1 ^d^	nd	nd	tr	nd	tr	1482.7 ± 11.7 ^p^
	Cheongja	111.2 ± 4.6 ^no^	99.5 ± 2.8 ^lm^	89.5 ± 3.6 ^hi^	266.1 ± 14.9 ^op^	138.2 ± 7.0 ^lm^	642.9 ± 14.6 ^mn^	nd	nd	nd	tr	nd	0.7 ± 0.0 ^s^	2150.9 ±36.0 ^j^

^1^ All values are expressed as the mean ± SD of triplicate determinations. Means in the same column by the same letter are not significantly different at the level of 0.05 by using Tukey’s HSD-ANOVA multiple range test. Content expressed μg of each component equivalents per g of dried weight. ^2^ nd, not detected. ^3^ tr, trace. Din, daidzin; Gly, glycitin; Gin, genistin; MDin, malonyldaidzin; MGly, malonylglycitin; MGin, malonylgenitin; AcDin, acetyldaidzin; AcGly, acetylglycitin; AcGin, acetylgenistin; Dein, dadzein; Glein, glycitein; Gein, genistein.

**Table 2:** Comparisons of protein and oil contents in four categories based on Korean soybean usage.

Contents of Protein and Oil from Soybeans Categories in the Republic of Korea (%) ^1^					
Bean Paste Soybeans	Protein	Oil	Bean Sprout Soybeans	Protein	Oil
Shinpaldal 2	43.1 ± 2.7 ^ab^	21.2 ± 1.4 ^ab^	Eunha	44.0 ± 3.1 ^ab^	18.5 ± 1.6 ^jk^
Taekwang	38.9 ± 3.0 ^hi^	21.9 ± 0.8 ^ab^	Soho	38.7 ± 2.4 ^jk^	20.8 ± 0.7 ^ab^
Samnam	41.4 ± 2.4 ^bc^	20.2 ± 1.1 ^bc^	Myeongjunamul	38.2 ± 1.6 ^lm^	20.9 ± 0.9 ^ab^
Pokwang	39.3 ± 1.9 ^gh^	20.5 ± 1.9 ^ab^	Iksannamul	40.9 ± 2.8 ^cd^	22.3 ± 1.5 ^a^
Dankyeong	39.6 ± 2.1 ^fg^	21.0 ± 1.5 ^ab^	Sobaegnamul	40.1 ± 3.0 ^de^	20.0 ± 1.4 ^bc^
Jinpum 2	39.4 ± 2.6 ^kl^	19.5 ± 0.7 ^bc^	Bukwang	39.3 ± 1.5 ^de^	20.5 ± 1.7 ^ab^
Daewon	41.1 ± 3.2 ^cd^	21.5 ± 2.0 ^ab^	Anpyeong	41.7 ± 1.8 ^cd^	20.0 ± 2.1 ^de^
Jangwon	44.1 ± 2.7 ^ab^	19.5 ± 1.3 ^gh^	Dagi	38.9 ± 2.3 ^kl^	19.4 ± 0.6 ^hi^
Daehwang	44.1 ± 2.9 ^ab^	18.8 ± 1.6 ^ij^	Sorog	42.6 ± 2.0 ^ab^	18.8 ± 1.5 ^gh^
Daemang	41.9 ± 3.6 ^ab^	20.5 ± 0.9 ^ab^	Sowon	39.3 ± 2.9 ^ij^	20.8 ± 0.8 ^ab^
Malli	39.8 ± 1.5 ^hi^	20.5 ± 1.2 ^ab^	Sojin	36.8 ± 1.7 ^m^	22.3 ± 1.2 ^a^
Vegetable soybeans	Protein	Oil	Cooked-with-rice soybeans	Protein	Oil
Keunol	44.6 ± 2.7 ^ab^	17.0 ± 0.8 ^n^	Ilpumgeomjeong	42.5 ± 3.0 ^ab^	21.0 ± 1.0 ^ab^
Seokryangput	43.8 ± 1.8 ^ab^	20. 2± 0.6 ^bc^	Geomjeong 2	40.9 ± 2.4 ^ab^	20.6 ± 0.7 ^ab^
Hwaeomput	46.4 ± 2.2 ^ab^	18.0 ± 1.1 ^mn^	Geomjeongol	41.8 ± 3.3 ^bc^	19.3 ± 0.9 ^ef^
Hwasonput	45.7 ± 2.5 ^ab^	18.2 ± 1.4 ^lm^	Geomjeong 1	41.3 ± 2.7 ^de^	21.3 ± 1.^ab^
Danmi	43.1 ± 1.6 ^ab^	18.9 ± 1.0 ^fg^	Geomjeongsaeol	40.2 ± 1.9 ^ef^	20.1 ± 1.6 ^bc^
Shillog	43.9 ± 2.4 ^ab^	19.0 ± 2.2 ^ef^	Jinyul	42.0 ± 2.1 ^ab^	19.5 ± 1.1 ^cd^
Seonnog	44.8 ± 2.9 ^a^	19.6 ± 0.9 ^cd^	Heugcheong	40.4 ± 3.2 ^hi^	19.8 ± 0.7 ^bc^
Dajin	46.6 ± 2.0 ^ab^	18.2 ± 1.6 ^kl^	Seonheuk	43.5 ± 2.6 ^ab^	20.9 ± 1.3 ^ab^
			Cheongdu 1	43.6 ± 1.6 ^ab^	20.4 ± 1.9 ^bc^
			Geomjeong 3	43.9 ± 1.3 ^ab^	18.6 ± 2.1 ^hi^
			Cheongja	41.7 ± 2.8 ^ab^	21.0 ± 1.4 ^ab^

^1^ All values are expressed as mean ± SD of triplicate experiments on a dry-weight basis. The same letters are not significant at the 0.05 level of Tukey’s HSD-ANOVA multiple range tests.

## Data Availability

The data will be made available on request.

## Supplementary Materials

The following supporting information can be downloaded at: https://zenodo.org/records/15515329, Figure S1: 12 Positive ESI-TOF-MS spectra of 12 isoflavone derivatives, Table S1. Comparison of soybean cultivars with the radical scavenging and enzyme inhibition activities of four usage categories using positive controls, Table S2: Isoflavone profiles in the 50% methanol extract of soybean (cv. Sorog) by UPLC-ESI-Q-TOF-MS/MS analysis, Table S3. Cumulative value (%) of the component variance of metabolites from soybean of the four usages.

## Abbreviations

The following abbreviations are used in this manuscript:

ABTS	2,2-azinobis (3-ethyl-benzothiazoline-6-sulfonic acid) diammonium salt
DPPH	2,2-diphenyl-1-picrylhydrazyl
BHT	Butylated hydroxytoluene
HPLC	High performance liquid chromatography
UPLC-Q-TOF-MS/MS	Ultra-performance liquid chromatography coupled with quadrupole time-of-flight mass spectrometry

## References

[1] Jahan T., Huda M.N., Zhang K., He Y., Lai D., Dhami N., Quinet M., Ali M.A., Kreft I., Woo S.H. (2025). Plant secondary metabolites against biotic stresses for sustainable crop protection. Biotechnol. Adv..

[2] Jha Y., Macwan A.A., Ghanaim A.M., Mohamed H.I. (2024). Management of abiotic and biotic stresses by microbiome-based engineering of the rhizosphere. Biocatal. Agric. Biotechnol..

[3] Lahlali R., Taoussi M., Laasli S.E., Gachara G., Ezzouggari R., Belabess Z., Aberkani K., Assouguem A., Meddich A., Jarroudi M.E. (2024). Effects of climate change on plant pathogens and host-pathogen interactions. Crop Environ..

[4] Nurmilah S., Frediansyah A., Cahyana Y., Utama G.L. (2024). Biotransformation and health potential of isoflavones by microorganisms in Indonesian traditional fermented soy products: A review. J. Agric. Food Res..

[5] Shiade S.R.G., Zand-Silakhoor A., Fathi A., Rahimi R., Minkina T., Rajput V.D., Zulfiqar U., Chaudhary T. (2024). Plant metabolites and signaling pathways in response to biotic and abiotic stresses: Exploring bio stimulant applications. Plant Stress.

[6] Malu M., Chatterjee J., Choudhary D., Ramakrishna W., Kumar R. (2025). Biotic, abiotic, and genetic elicitors as a new paradigm for enhancing alkaloid production for pharmaceutical applications. S. Afr. J. Bot..

[7] Yin Q., Feng Z., Ren Z., Wang H., Wu D., Jaisi A., Yang M. (2025). Integrative physiological, metabolomic and transcriptomic insights into phenylpropanoids pathway responses in *Nicotiana tabacum* under drought stress. Plant Stress.

[8] Samanta S., Seth C.S., Roychoudhury A. (2024). The molecular paradigm of reactive oxygen species (ROS) and reactive nitrogen species (RNS) with different phytohormone signaling pathways during drought stress in plants. Plant Physiol. Biochem..

[9] Chen Y., Wang Y., Chen J., Tang H., Wang C., Li Z., Xiao Y. (2020). Bioprocessing of soybeans (*Glycine max* L.) by solid-state fermentation with *Eurotium cristatum* YL-1 improves total phenolic content, isoflavone aglycones, and antioxidant activity. RSC Adv..

[10] Choi Y.-M., Yoon H., Shin M.-J., Lee Y., Hur O.S., Lee B.C., Ha B.-K., Wang X., Desta K.T. (2021). Metabolite contents and antioxidant activities of soybean (*Glycine max* (L.) Merrill) seeds of different seed coat colors. Antioxidants.

[11] Solimine J., Garo E., Wedler J., Rusanov K., Fertig Q., Hamburger M., Atanassov I., Butterweck V. (2016). Tyrosinase inhibitory constituents from a polyphenol enriched fraction of rose oil distillation wastewater. Fitoterapia.

[12] Wang L., Zhu X., Liu H., Sun B. (2025). Medicine and food homology substances: A review of bioactive ingredients, pharmacological effects and applications. Food Chem..

[13] Waqas M.K., Akhtar N., Mustafa R., Jamshaid M., Kan H.M.S., Murtaza G. (2015). Dermatological and cosmeceutical benefits of *Glycine max* (soybean) and its active components. Acta Pol. Pharm. Drug Res..

[14] Zheng Z.P., Tan H.Y., Chen J., Wang M. (2013). Characterization of tyrosinase inhibitors in the twigs of *Cudrania tricuspidata* and their structure-activity relation study. Fitoterapia.

[15] Harada K., Fukusaki E. (2009). Profiling of primary metabolite by means of capillary electrophoresis-mass spectrometry and its application for plant science. Plant Biotechnol..

[16] Cardoso C.C., Mendes B.M.O., Pasa V.M.D. (2018). Production and characterization of cold-flow quality biofuel from soybean oil using different alkyl and benzyl alcohols. J. Environ. Chem. Eng..

[17] Chitisankul W.T., Itabashi M., Son H., Takahashi Y., Ito A., Varanyanond W., Tsukamoto C. (2021). Soyasaponin composition complexities in soyfoods relating nutraceutical properties and undesirable taste characteristics. LWT-Food Sci. Technol..

[18] Qin P., Wang T., Luo Y. (2022). A review on plant-based proteins from soybean: Health benefits and soy product development. J. Agric. Food Res..

[19] Lee J.H., Seo E.Y., Lee Y.M. (2023). Comparative investigation on variations of nutritional components in whole seeds and seed coats of Korean black soybeans for different crop years and screening of their antioxidant and anti-aging properties. Food Chem. X.

[20] Chu H.-N., Lee S.-J., Wang X., Lee S.-H., Yoon H.-M., Hwang Y.-J., Jung E.-S., Kwon Y., Wee C.-D., Jang K.-A. (2021). A correlation study on in vitro physiological activities of soybean cultivars, 19 individual isoflavone derivatives, and genetic characteristics. Antioxidants.

[21] Alu’datt M.H., Rababah T., Ereifej K., Alli I. (2013). Distribution, antioxidant, and characterization of phenolic compounds in soybeans, flaxseed and olives. Food Chem..

[22] Lee H., Yang J.Y., Ra J.E., Ahn H.J., Lee M.J., Kim H.Y., Song S.Y., Kim D.H., Lee J.H., Seo W.D. (2023). Elucidation of phenolic metabolites in wheat seedlings (*Triticum aestivum* L.) by NMR and HPLC-Q-Orbitrap-MS/MS: Changes in isolated phenolics and antioxidant effects through diverse growth times. Food Chem. X.

[23] Rahman M.J., Ambigaipalan P., Shahidi F. (2018). Biological activities of camelina and sophia seeds phenolics: Inhibition of LDL oxidation, DNA damage, and pancreatic lipase and α-glucosidase activities. J. Food Sci..

[24] Hwang C.E., Kim S.C., Kim D.H., Lee H.Y., Suh H.K., Cho M.C., Lee J.H. (2021). Enhancement of isoflavone aglycone, amino acid, and CLA contents in fermented soybean yogurts using different strains: Screening of antioxidant and digestive enzyme inhibition properties. Food Chem..

[25] Yin X., Ren Z., Jia R., Wang X., Yu Q., Zhang L., Liu L., Shen W., Fang Z., Liang J. (2024). Metabolic profiling and spatial metabolite distribution in wild soybean (*G. soja*) and cultivated soybean (*G. max*) seeds. Food Chem. X.

[26] Chang S.K.C., Tan Y. (2024). Mass yields, antioxidant and anti-DU145 prostate cancer cell proliferation properties of prosoy soymilk as affected by extraction methods and cooking. Antioxidants.

[27] Erba D., Casiraghi M.C., Martinez-Conesa C., Goi G., Massaccesi L. (2012). Isoflavone supplementation reduces DNA oxidative damage and increases O-β-N-acetyl-D-glucosaminidase activity in healthy women. Nutr. Res..

[28] Liu W.T., Huang C.L., Liu R., Yang T.C., Lee C.L., Tsao R., Yang W.J. (2023). Changes in isoflavone profile, antioxidant activity, and phenolic contents in Taiwanese and Canadian soybeans during tempeh processing. LWT-Food Sci. Technol..

[29] Sohn S.I., Pandian S., Oh Y.J., Kang H.J., Cho W.S., Cho Y.S. (2021). Metabolic engineering of isoflavones: An updated overview. Front. Plant Sci..

[30] Spréa R.M., Finimundy T.C., Calhelha R.C., Pires T.C.S.P., Prieto M.A., Amaral J.S., Barros L. (2024). Comprehensive analysis of soybean (*Glycine max* L.) by-products: Nutritional value, phenolic composition, and bioactive properties. Food Biosci..

[31] Jung S.J., Shin D.H. (2024). Gochujang, a Korean traditional fermented soybean product: History, preparation and functionality. J. Ethn. Foods.

[32] Kim S.H., Ko J., Kwon D.Y. (2023). Jang, Korean fermented soybean product, the result of endeavors of ancients for the best taste of Korean diet. J. Ethn. Foods.

[33] Kim J.A., Hong S.B., Jung W.S., Yu C.Y., Ma K.H., Gwag J.G., Chung I.M. (2007). Comparison of isoflavones composition in seed, embryo, cotyledon and seed coat of cooked-with-rice and vegetable soybean (*Glycine max* L.) varieties. Food Chem..

[34] Kim D.H., Yang W.T., Cho K.M., Lee J.H. (2020). Comparative analysis of isoflavone aglycones using microwave-assisted acid hydrolysis from soybean organs at different growth times and screening for their digestive enzyme inhibition and antioxidant properties. Food Chem..

[35] Lee J.H., Hwang C.E., Cho E.J., Song Y.H., Kim S.C., Cho K.M. (2018). Improvement of nutritional components and in vitro antioxidative properties of soy-powder yogurts using *Lactobacillus plantarum*. J. Food Drug Anal..

[36] Lee H.Y., Cho D.Y., Kim D.H., Park J.H., Jeong J.B., Jeon S.H., Lee J.H., Ko E.J., Cho K.M., Lee J.H. (2024). Examining the alterations in metabolite constituents and antioxidant properties in mountain-cultivated ginseng (*Panax ginseng* C.A. Meyer) organs during a two-month maturation period. Antioxidants.

[37] Hong J.L., Qin X.Y., Shu P., Wang Q., Zhou Z.F., Wang G.K., Lin N.B., Wang Q., Qin M.J. (2011). Comparative study of isoflavones in wild and cultivated soybeans as well as bean products by high-performance liquid chromatography coupled with mass spectrometry and chemometric techniques. Eur. Food Res. Technol..

[38] Chong J., Wishart D.S., Xia J. (2019). Using MetaboAnalyst 4.0 for comprehensive and integrative metabolomics data analysis. Curr. Protoc. Bioinform..

[39] Chen O., Wang X., Yuan X., Shi J., Zhang C., Yan N., Jing C. (2021). Comparison of phenolic and flavonoid compound profiles and antioxidant and α-glucosidase inhibition properties of cultivated soybean (*Glycine max*) and wild soybean (*Glycine soja*). Plants.

[40] Kim I.-S. (2021). Current perspectives on the beneficial effects of soybean isoflavones and their metabolites for humans. Antioxidants.

[41] Seal T., Pillai B., Chaudhuri K. (2022). DNA damage preventive activity of wild edible plants. Food Chem. Adv..

[42] Zheng Y., Zhang R., Huang F., Cheng L., Xu L., Jiac X. (2023). α-Glucosidase inhibitors derived from black soybean and their inhibitory mechanisms. LWT-Food Sci. Technol..

[43] Bodurlar Y., Caliskan M. (2022). Inhibitory activity of soybean (*Glycine max* L. Merr.) Cell Culture Extract on tyrosinase activity and melanin formation in alphamelanocyte stimulating Hormone-Induced B16-F10 melanoma cells. Mol. Biol. Rep..

